# Insights into dynamics and gating properties of T2SS secretins

**DOI:** 10.1126/sciadv.adg6996

**Published:** 2023-10-04

**Authors:** Brice Barbat, Badreddine Douzi, Geneviève Ball, Mathilde Tribout, Khalid El Karkouri, Christine Kellenberger, Romé Voulhoux

**Affiliations:** ^1^ LCB-UMR7283, CNRS, Aix Marseille Université, IMM, Marseille, France.; ^2^ Université de Lorraine, INRAE, DynAMic, Nancy, F-54000 France.

## Abstract

Secretins are outer membrane (OM) channels found in various bacterial nanomachines that secrete or assemble large extracellular structures. High-resolution 3D structures of type 2 secretion system (T2SS) secretins revealed bimodular channels with a C-module, holding a conserved central gate and an optional top gate, followed by an N-module for which multiple structural organizations have been proposed. Here, we perform a structure-driven in vivo study of the XcpD secretin, which validates one of the organizations of the N-module whose flexibility enables alternative conformations. We also show the existence of the central gate in vivo and its required flexibility, which is key for substrate passage and watertightness control. Last, functional, genomic, and phylogenetic analyses indicate that the optional top gate provides a gain of watertightness. Our data illustrate how the gating properties of T2SS secretins allow these large channels to overcome the duality between the necessity of preserving the OM impermeability while simultaneously promoting the secretion of large, folded effectors.

## Teaser

Structure-based exploration of secretins opens the way to better understand these giant portals of the bacterial envelope.

## INTRODUCTION

Recent high-resolution three-dimensional (3D) structures of bacterial secretins have revealed the structural organization of this outer membrane (OM) portal of bacterial nanomachines specialized in the translocation of large structures (*1*–*4*). Secretins mediate the cell surface exposure of type 4 pili (T4P) or needle complexes of type 3 secretion systems (T3SSs) and the secretion of large structures such as bacteriophages in filamentous phage extrusion systems (FPESs) or fully folded effectors in type 2 secretion system (T2SS) (*5*). This out-of-gauge property is ensured by the ability of secretins to assemble into giant oligomers defining a large internal cavity inserted in the OM via its C-terminal extremity, while its N terminus extends in the periplasmic space, up to the inner membrane (IM).

**Fig. 1.**
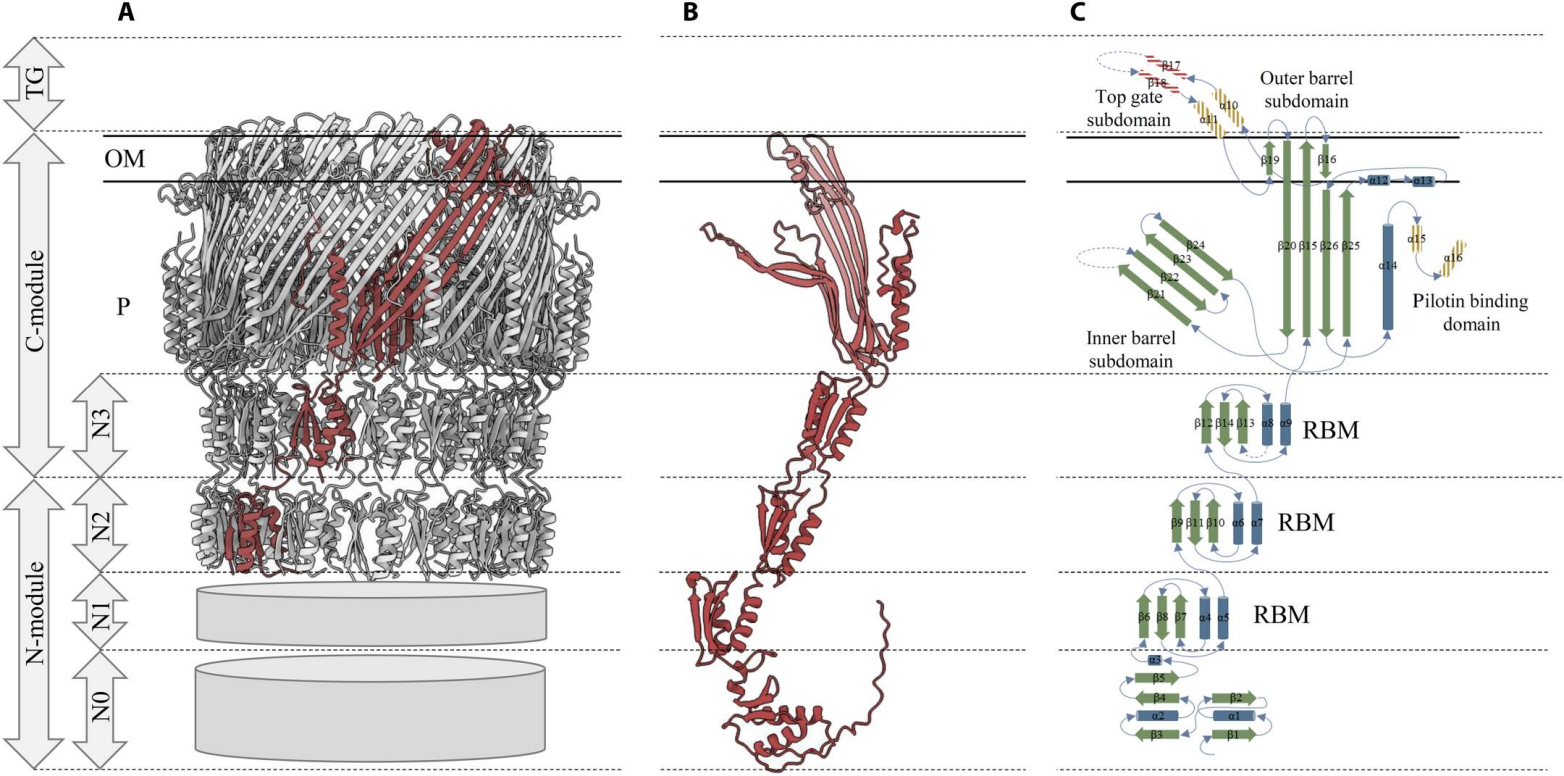
Structural organization of T2SS secretins. (**A**) Near atomic–resolution cryo-EM structure of the *Klebsiella-type* XcpD secretin from *P. aeruginosa* [Protein Data Bank (PDB): 5WLN] (*10*). The C-module and N2 domain are fully resolved, while the N0 and N1 domains are not. (**B**) AlphaFold prediction model (*48*) of the full-length XcpD monomer (UniprotKB, P35818) allows the visualization of the N1 and N0 domain organized as seen in (*8*). (**C**) Topological diagram of secondary structural elements of a canonical T2SS secretin monomer. Hatched areas correspond to not conserved secondary structures encoding for top gate and pilotin binding domain [adapted from (*4*)]. OM, outer membrane; P, periplasm; RBM, ring-building motif.

Secretins are bimodular complexes including a well-conserved OM C-module, followed by a variable periplasmic N-module composed of several superposed ring-like structures, whose number depends on the transport machine (Fig. 1A) (*6*). The C-module of secretins is constituted by the N3 ring-building motif (RBM) (*7*) and the pore-forming or secretin domain, ended by an optional S domain (*5*). The structural organization of C-modules is well defined in the recently reported near-atomic 3D structures (*6*). It presents a pentadecameric (C15) symmetry for T2SS, T3SS, and FPES members (*1*, *3*, *4*, *8*–*16*), whereas T4P secretins are primarily tetradecameric (C14) (*2*, *17*), although different stoichiometries have been reported for a given secretin C-module (*2*, *4*). The pore-forming domain is singularly organized into a giant 56 to 60 stranded double-walled β barrel, where each of the 14 to 15 subunits contributes four β strands to the inner barrel and four β strands to the outer barrel (Fig. 1, B and C). In the inner barrel, the strand extremities bent perpendicularly to the barrel’s wall and converged toward the center of the cavity to partially obturate the channel. This structural feature, named central gate, is clearly visible in cryo–electron microscopy (cryo-EM) images of isolated particles and in cryo-tomography images of membrane-embedded secretins (*18*). The gating nature of the central gate is supported by several observations, including the finding that point mutations in central gate or in a glycine residue located at one bending point of the central gate’s β strands, lead to leaky phenotypes, such as antibiotic sensitivity or bile salt permeability (*4*, *19*). As shown by the recent 3D structure determination of a T3SS secretin in an open conformation (*12*), these small and flexible glycine residues would allow the rotation of the central gate, which would flip upward and come close to the β barrel wall to accommodate the secreted or assembled structures. Similarly, the insertion of two cysteines (Cys) in the inner barrel of the T4P secretin PilQ from *Vibrio cholerae* resulted in a defect of functionality, again highlighting the necessary flexibility of this region (*17*).

With the release of secretin structures, the presence of an additional apical domain called top gate (or cap gate) was reported in a subclass of T2SS secretins named *Vibrio*-type (Fig. 1C) (*4*, *9*, *20*). The top gate protrudes above the OM surface in a conical shape, formed by the convergence of 15 pairs of antiparallel β strands whose connecting loops are relatively loose, suggesting a dynamic structure similar to the central gate. The inner diameter of the top gate defines a much narrower opening compared to the large open-edged β barrels in the top gate–free *Klebsiella*-type subclass (*9*). Given the directionality of effector transport in T2SS, the top gate should rotate outward to open and release the substrate in the extracellular milieu. The systematic poor resolution reported at the center of both top gate and central gate may reflect flexibility, consistent with their transient opening to allow the passage of the effectors.

The universal C-module of the secretins is extended in the periplasm by the N-module, which is nanomachine specific. This latter fits into the lower continuity of the C-module and contributes to a large periplasmic chamber, which is known to dock onto the IM partners of the secretin (*1*, *12*, *17*, *21*–*23*). The N-module is a combination of distinct domains connected via short linkers, whose number depends on the transport machine (Fig. 1) (*6*). All N-modules bear an N0 domain at their N terminus that is either followed by two N2 and N1 or only one N1 RBM domain(s) in T2SS and T3SS secretins, respectively. However, in contrast to well-defined C-modules, the low resolution of N-modules from isolated secretin particles visualized by cryo-EM does not allow an accurate positioning and numbering in the global structure (Fig. 1) (*1*, *3*, *4*, *10*, *17*). This limitation is regrettable because of the recurrent symmetry conflicts observed between C-modules of T3SS and T2SS secretins and their IM partners, which present incompatible 15:24 and 15:12 stoichiometries, respectively (*8*, *13*). This low cryo-EM resolution of N-modules, which becomes even more pronounced toward the N terminus, is probably due to the absence of a stabilizing partner(s). Much better resolution was obtained for this region when secretins are in complex with their IM partners (*8*, *13*–*15*). For the T3SS InvG secretin, an additional 16th N-module subunit is seen in the presence of its IM partner PrgH, leading to a compatible InvG_16_:PrgH_24_ interface, reinforced by eight additional PrgH subunits packing around and under the β sheet (*13*, *15*). This hexadecameric organization of the N-module, also observed for MxiD in *Shigella flexneri*, remains to be proven in vivo.

Chernyatina and Low (*8*) solved the structure of the T2SS secretin PulD by cryo-EM in complex with its partner PulC, observing that 15 PulD (with N0 domains arranged in a face-to-back C1 symmetry) interact with 12 PulC. This suggests that the 15:12 stoichiometry conflict is located between the secretin and its partner, but no mechanism has been proposed to accommodate such a symmetry mismatch. In contrast, we reported that the isolated N-module of the T2SS secretin XcpD spontaneously self-assembles into a hexamer of dimers, adopting a C12 stoichiometry with N0 domains that are arranged in a different face-to-face C2 symmetry (*21*, *23*). Hay *et al.* (*10*) proposed the existence of a pentadecameric XcpD secretin where three N-modules are displaced away from the XcpC/XcpD interface, leading to the formation of a metastable C15:N12 arrangement, thus overcoming the stoichiometry conflict within the secretin. This important reorganization between C- and N-modules, still to be confirmed in vivo, is supported by the long unstructured density, systematically observed at this interface and thus leaving enough space to accommodate this structural flexibility (Fig. 1A) (*4*).

All secretins studied so far harbor an N0 domain at their N-terminal extremity, physically involved in the interaction with the IM partners (*22*, *24*, *25*). The 3D structure organization of N0 domain was first determined for isolated N-modules, under monomeric or dimeric forms, in complex or not with their IM partners (*13*, *18*, *22*, *23*, *26*, *27*). Whereas N0 domains present a similar TonB-dependent transduction domain fold (*27*), their oligomeric organization appears to be variable in terms of stoichiometry not only between but also within secretin families. Inside the T3SS secretin family, for example, pentadecameric and hexadecameric stoichiometries have been proposed for the N0 domain (*12*, *13*). For T2SS secretins, pentadecameric and dodecameric stoichiometries were reported, with two different structural arrangements of their N-modules into face-to-back C1 or face-to-face C2 symmetries (*8*, *21*). Whether these two conformations of N0 domains simultaneously or sequentially coexist in T2SS secretins during the secretion process remains to be determined.

In the present study, we used several in vivo assays to explore the structure-based dynamics and gating properties of the T2SS secretin XcpD. Our data (i) reveal an additional flexibility point at the N0 interface, reconciling previous conflicting structural arrangements; (ii) validate the in vivo existence of a dynamic and specific central gate, allowing the secretion of large exoproteins while guaranteeing the sealing of the bacterial envelope; (iii) attribute a nonspecific gain of watertightness function to the optional top gate; and (iv) highlight the genomic and phylogenetic diversity of the apical extremity of T2SS secretins.

## RESULTS AND DISCUSSION

### A dynamic face-to-face interface between N0 domains in the XcpD secretin exists in vivo

**Fig. 2.**
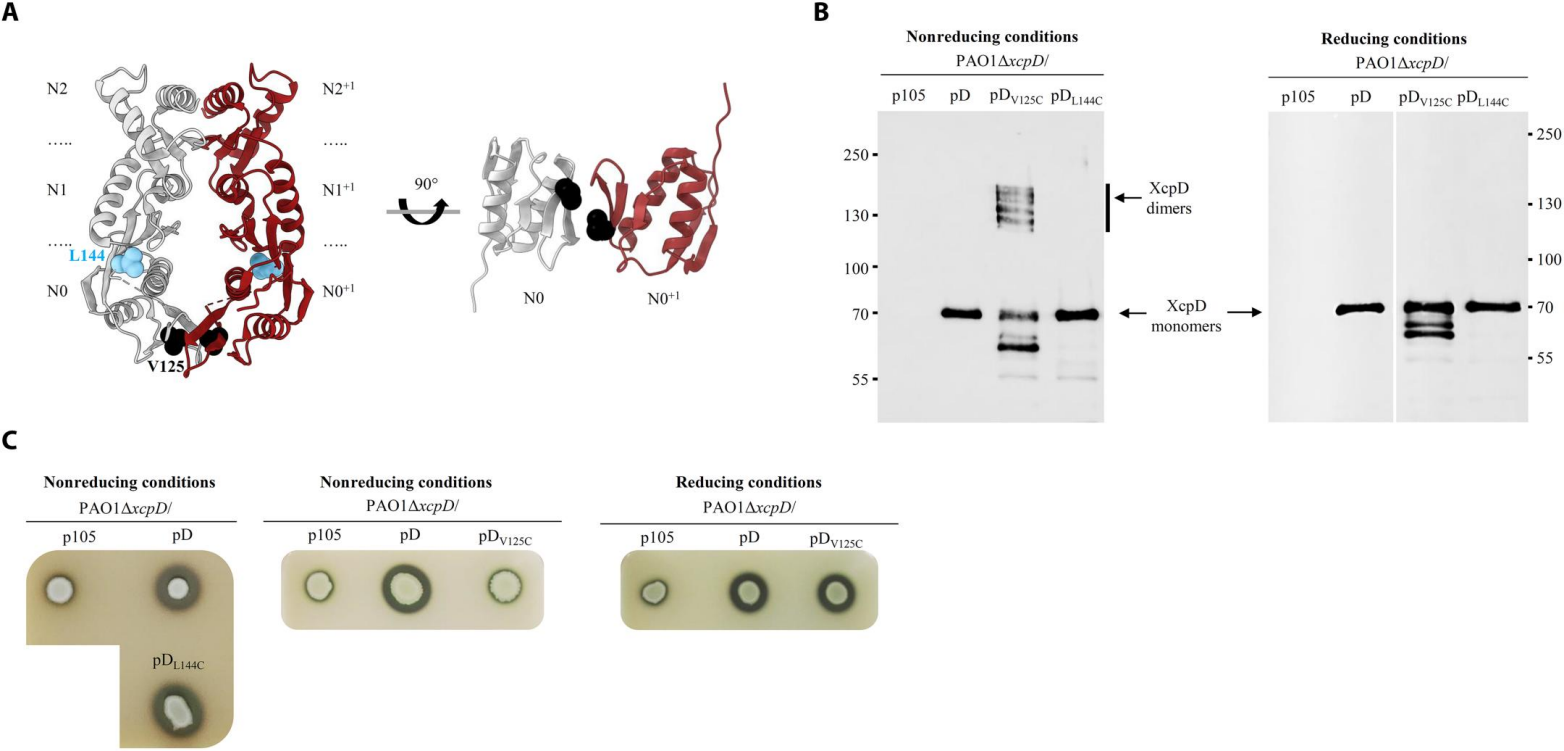
Structure/function analysis of the intradimer interface of the XcpD N-module suggests N0 flexibility. (**A**) Side and bottom views of an XcpD N-module homodimer (PDB: 3EZJ). In each monomer, the N2, N1, and N0 subdomains are indicated, as well as the amino acid V125 (black spheres) and L144 (blue spheres), which were submitted to Cys substitution. (**B**) Immunoblotting of OM protein samples under normal nonreducing or βME-induced reducing conditions with anti-V5 antibody. The Δ*xcpD* mutant in strain PAO1 was complemented by the empty vector (p105), the WT *xcpD* (pD), or the mutant *xcpD*_V125C_ (pD_V125C_), and *xcpD*_L144C_ genes (pD_L144C_). Molecular mass markers in kilodaltons are indicated on the left or right sides. The quality of the OM fractionation has been checked by the absence of periplasmic and IM control components (fig. S1). (**C**) In vivo plate secretion assay under nonreducing or reducing conditions (5 mM DTT). The activity of proteases secreted by the T2SS was assayed on skim milk agar plates by measuring the halo of milk degradation around bacterial colony, revealing the activity of the Xcp-dependent exoproteases and, consequently, the secretion efficiency of the corresponding T2SS secretin.

The N-module of T2SS secretins is constituted by two N2 and N1 RBM domains and a TonB-dependent N-terminal N0 domain whose quaternary structural organization is still a matter of debate. While the cryo-EM analysis of full-length *Klebsiella oxytoca* PulD secretin in complex with partners indicated an N-module with face-to-back C1 symmetry (*8*), the isolated N-module of the *Pseudomonas aeruginosa* XcpD secretin was reported to assemble spontaneously into a hexamer of dimers, where monomers are oriented in a face-to-face C2 symmetry (Fig. 2A) (*21*). Although these organizations seem incompatible at first glance, they can also reflect two dynamic states of the secretin during the secretion process. However, this would require flexibility at the N0/N0^+1^ interface, which is yet to be demonstrated.

To validate in vivo the XcpD intradimer N0 interface and evaluate its flexibility, we applied the previously developed in vivo reversible Cys cross-linking (rCys-XL) strategy (*21*) after selecting the valine residue at position 125 of XcpD as a target residue. In the C2 face-to-face model, V125 residues in two adjacent N0 subunits are predicted to be at a distance compatible with the formation of a disulfide bridge (Fig. 2A). We therefore engineered a Cys substitution of XcpD V125 and analyzed the corresponding full-length XcpD_V125C_ variant in vivo for cross-linking and secretion capacity. XcpD_V125C_ was produced by transcomplementation in the *P. aeruginosa* PAO1 strain deleted for its chromosomal *xcpD* gene (Δ*xcpD*). Western blot analysis, under nonreducing conditions, of OM protein samples of the wild-type (WT) XcpD and the XcpD_V125C_ Cys variant, produced under natural nonreducing conditions, showed that full-length XcpD_V125C_ forms dimers in the OM, which are absent in the XcpD control (Fig. 2B, left). The partial degradation of XcpD_V125C_ in the OM fraction induced the formation of dimers of different sizes. It is important at this stage to mention that only correctly localized secretin variants at the OM (fig. S1), under oligomeric form and resistant to 4 M urea (fig. S2), alike the WT, were considered in this study. This frees us from any detection of aggregates or complexes arising from secretins accumulated in the IM, stuck in transit to the OM, or not properly integrated into the OM (fig. S1). The strict dependency of these dimers on the C125-C125 disulfide bridge is demonstrated by its dissociation when β-mercaptoethanol (βME)–reducing agent is added to the OM sample (Fig. 2B, right). The C125-C125 disulfide bridge confirms the existence in vivo of the N0/N0^+1^ face-to-face interface in the OM-inserted secretin.

Functional consequences on T2SS secretion were evaluated by extracellular Xcp T2SS effector secretion (fig. S3) and plate secretion assay on tryptic soy agar (TSA)–skim milk plates (Fig. 2C, middle), reporting the extracellular activity of the Xcp proteases (*21*), whose secretion is XcpD dependent. These experiments revealed that the bridging of XcpD_V125C_ dimers leads to marked reduction of extracellular protease activity, indicating that the interface needs to be flexible to allow protein secretion through the T2SS secretin channel. The direct and specific correlation between disulfide bridge formation at position 125 of XcpD and T2SS inhibition was confirmed by the recovery of protease secretion, which was observed upon the reduction of the disulfide bridge. This was achieved by growing bacteria in the presence of the reducing agent dithiothreitol (DTT) (Fig. 2C, right). As negative control, we showed that the XcpD_L144C_ variant harboring a Cys residue at a position, not predicted to be at disulfide bridge distance from the same position of an adjacent N0 subunit in either face-to-face or face-to-back conformations, does not form disulfide bridge nor affects secretion under natural nonreducing conditions (Fig. 2). Together, our in vivo data (i) confirm that the N0 domain of the OM-inserted XcpD secretin displays the C2 face-to-face organization, which was previously proposed (*21*, *23*), and (ii) show that this C2 face-to-face organization is flexible, leaving the possibility for alternative conformations (*8*, *21*).

### In vivo existence and flexibility requirement of the XcpD central gate

**Fig. 3.**
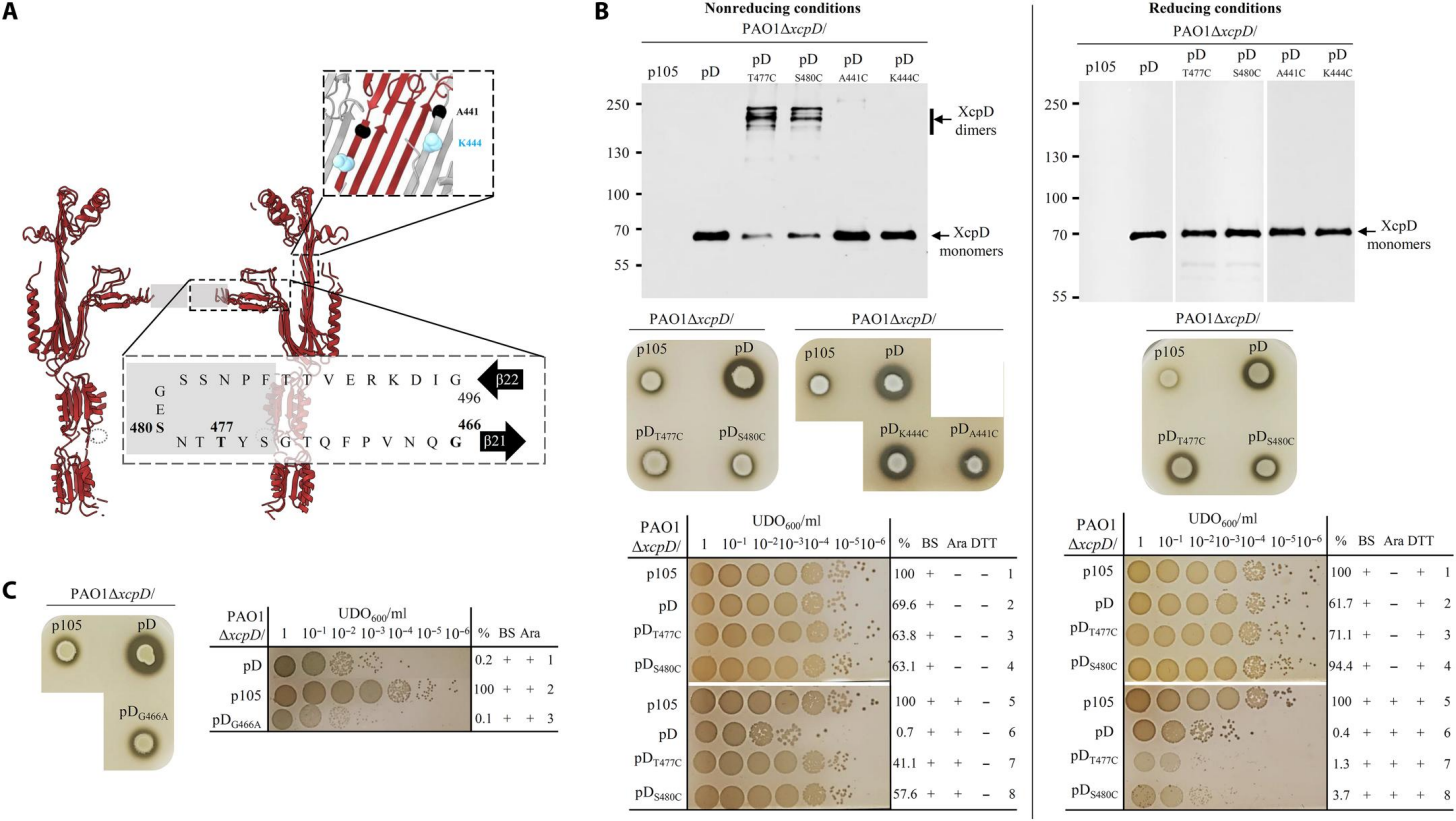
Structure/function analysis of the central gate in XcpD shows its flexibility requirement for secretion and watertightness. (**A**) Section view of the cryo-EM 3D structure of XcpD (PDB: 5WLN). XcpD central gate is constituted by 15 horizontal loops from each monomer, corresponding to amino acids 466 to 496—between β21 and β22 (see Fig. 1C)—whose extremities (gray box) are poorly defined in the structure. The amino acids G466 (pivot), T477 and S480 (central gate), and A441 and K444, which were submitted to mutagenesis, are highlighted. (**B**) Functional investigation of point substitutions T477C, S480C, A441C, and K444C in XcpD under nonreducing (left) or reducing (right) conditions. Top: Immunoblotting of OM protein samples under nonreducing or reducing conditions, with anti-V5 antibody. The Δ*xcpD* mutant in strain PAO1 was complemented by the empty vector (p105), the WT *xcpD* gene (pD), or the mutant *xcpD_T477C_* (pD_T477C_), *xcpD_S480C_* (pD_S480C_), *xcpD_A441C_* (pD_A441C_), and *xcpD_K444C_* (pD_K444C_) genes. Molecular mass markers in kDa are indicated on the left. The quality of the OM fractions has been checked (see fig. S1). Middle: In vivo plate secretion assay, performed as in Fig. 2C but with 7 mM DTT for the reducing conditions. Bottom: Bile salt sensitivity assay on plate under nonreducing or reducing conditions. Growth of the different strains was tested after spotting serial 10-fold dilutions on 2% bile salt (BS) agar plates, supplemented (+) or not (−) with 0.05% arabinose (Ara) and/or 3 mM DTT. Quantitative data of three independent biological replicates are presented as a mean percentage (%) relative to the reference strain PAO1ΔD/p105. (**C**) Left: Functional investigation of point mutant XcpD_G466A_ in the glycine pivot by in vivo plate secretion assay. Right: Bile salt sensitivity assay on plate of the XcpD_G466A_ variant performed and presented as in (B) (bottom).

One of the most remarkable features revealed by high-resolution cryo-EM images of isolated (*3*, *4*) or membrane-embedded (*18*) secretins is the presence, at the lumen of the C-module, of a well-defined horizontal closure that was called central gate. The central gate is formed by the convergence of 15 horizontal loops, which link two β sheets arising from the inner β barrel of secretin C-module (Fig. 3A). On the basis of sequence homology, the central gate of T2SS secretins occurs between two conserved glycine residues called pivots (Fig. 3A and fig. S4) (*4*). In our quest to determine the biological relevance of the structural determinants of secretins revealed by cryo-EM, we wanted to confirm the in vivo existence of the central gate and evaluate its flexibility during the secretion process. Thanks to the precise assignation of the amino acids involved in its formation, we were able to apply the rCys-XL strategy using specific residues. Hence, two independent Cys substitutions were engineered at positions 477 and 480 of XcpD, located at the tip of the loop involved in central gate formation and therefore predicted to be positioned at disulfide bridge distance from the same amino acid of a neighboring subunit (Fig. 3A). We analyzed each XcpD_T477C_ and XcpD_S480C_ Cys variant for its in vivo cross-linking capacities and functional properties by transcomplementation in a Δ*xcpD* mutant. OM protein samples of the WT XcpD and the two Cys variants were analyzed under nonreducing and reducing conditions (Fig. 3B, top). As shown by Western blotting experiments, higher–molecular weight oligomers of XcpD_T477C_ and XcpD_S480C_ Cys variants were specifically detected under natural nonreducing conditions (Fig. 3B, top left). These oligomers, of about twice the size of the monomers, are certainly dimers of XcpD resulting from the disulfide bridge formation between the unique free Cys of two adjacent or facing monomers. Dimer formation of XcpD_T477C_ and XcpD_S480C_ in OM samples validates the existence of the predicted central gate in fully assembled secretins inserted in the OM (figs. S1 and S2).

The functional consequences of disulfide bridge formation at the heart of the central gate on T2SS secretion was evaluated by extracellular T2SS effector deficiency and plate secretion assay on skim milk plates. The lack of Xcp T2SS effector secretion (fig. S3) and T2SS degradation halo around the complemented strains producing XcpD_T477C_ and XcpD_S480C_ variants (Fig. 3B, middle left) grown under nonreducing condition indicates a defect of Xcp-dependent secretion, probably due to the blockage of the central gate in the “closed” conformation imposed by the disulfide bridge. This demonstrates that the flexibility of the central gate is essential for the proper functioning of the secretin, such as for the N0 domain above. The direct implication of the artificial disulfide bridges on the blocking of the secretion process was demonstrated by restoring secretion under reducing conditions in the presence of the DTT (Fig. 3B, middle right). As negative control, we showed that two XcpD variants harboring a Cys residue at positions 441 and 444 in the C domain, not predicted to be at disulfide bridge distance from the same position of an adjacent subunit, do not form disulfide bridges nor affect secretion under natural nonreducing conditions (Fig. 3B). Our data therefore demonstrate that the central gate of bacterial T2SS secretins is specifically subjected to dynamic constraints during the secretion process. Dynamics in this region of the secretin is supported by the poor resolution of the central gate centers formed by the extremity of each 15 loops in all cryo-EM maps (Fig. 3A) (*10*).

Considering the detrimental effect of disulfide bridge formation in the central gate on secretin functionality, we tested its impact on watertightness properties by measuring the secretin-mediated permeability to bile salts. A direct correlation between bile salt sensitivity and secretin permeability has already been reported (*4*, *19*). We beforehand observed that overproduction of the WT XcpD triggers an increased bile salt sensitivity (Fig. 3B, bottom, and fig. S5, A to D, compare lines 2 and 6), probably due to the enrichment of isolated forms in the OM. We then determined the sensitivity to bile salt of *P. aeruginosa* strains producing XcpD_T477C_ and XcpD_S480C_ central gate variants under nonreducing and reducing conditions (Fig. 3B, bottom, and fig. S5, B and D). We found that, unlike the WT XcpD, the overproduction of the XcpD_T477C_ and XcpD_S480C_ variants under nonreducing conditions did not induce significant increase in bile salt sensitivity despite similar localization in the OM (Fig. 3B, bottom left, and fig. S5B compare lines 6, 7, and 8). Furthermore, our results reveal that under reducing conditions, the two central gate variants present a similar bile salt sensitivity phenotype than the WT (Fig. 3B, bottom right, and fig. S5D compare lines 6, 7, and 8). These data indicated that the nonfunctional conformation of the central gate imposed by the interdimer disulfide bridge constitutes a barrier to the passive entry of extracellular molecules, conferring sealing properties to the central gate. This phenotype possibly reflects a physiological closed conformation transiently adopted by the central gate of T2SS secretins during the secretion process. Such a specific sealing property is consistent with the need for a large channel to allow the passage of the typical large T2SS folded effectors. Considering the relatively small diameter of the cholic acid molecule that constitutes bile salts, we estimate that the central gate orifice under its disulfide bridged closed conformation is less than 15 Å wide, thereby validating the positioning of residues 477 or 480 with respect to the same residue of a neighboring subunit at a distance compatible with the formation of a disulfide bridge.

The glycine pivot residues at the base of the two β strands forming the 15 loops of the central gate (Fig. 3A) were suspected to be involved in central gate flexibility by Yan *et al.* (*4*) who showed that substitution of glycine-466 into alanine leads to a partial opening of the central gate. We examined the in vivo impact of this substitution on XcpD functionality and watertightness by performing secretion and bile salt sensitivity assays. Data indicate that the XcpD_G466A_ central gate–altered variant is not functional (Fig. 3C, left, and fig. S3), while it remains highly sensitive to bile salts (Fig. 3C, right, and fig. S5E, compare lines 1 and 3). This agrees with the proper targeting and oligomerization of the XcpD_G466A_ variant into the OM (figs. S1 and S2). These results indicate that it is possible to uncouple effector and bile salt transport, suggesting that these two transport processes are mediated by different mechanisms. We propose that while bile salt transits through T2SS secretin via a passive process, requiring a slight opening of the gate, the folded effectors only pass through a fully functional central gate via an active secretion process, which remains to be precisely determined.

Because flexibility was also reported at N0 level of the secretin, we tested the involvement of this dynamic region in bile salt sensitivity. Sensitivity to bile salts was therefore compared between PAO1 Δ*xcpD* strains producing the WT or the XcpD_V125C_ variant under nonreducing and reducing conditions. We observed that, in contrast to the central gate variants, the XcpD_V125C_ variant present a WT bile salt sensitivity in both conditions (fig. S6, compare lines 5 and 6 on both left and right panels). This observation, which precludes any impact of the XcpD_V125C_ degradation products on the bile salts sensitivity phenotype, indicates that locking the XcpD N0 domain under C2 closed conformation leaves a sufficient aperture for the passage of small molecules such as bile salts. This observation agrees with the ~45-Å internal diameter of the XcpD N-module under closed conformation proposed by Douzi and colleagues (*21*). This is too narrow to accommodate the 55-Å-wide secreted effector CbpD (*28*) or the endopilus with an external diameter of at least 60 Å (*29*, *30*), but it is large enough to allow passive diffusion of bile salt molecules (~13 Å).

### The optional top gate increases secretin impermeability without imposing substrate specificity

**Fig. 4.**
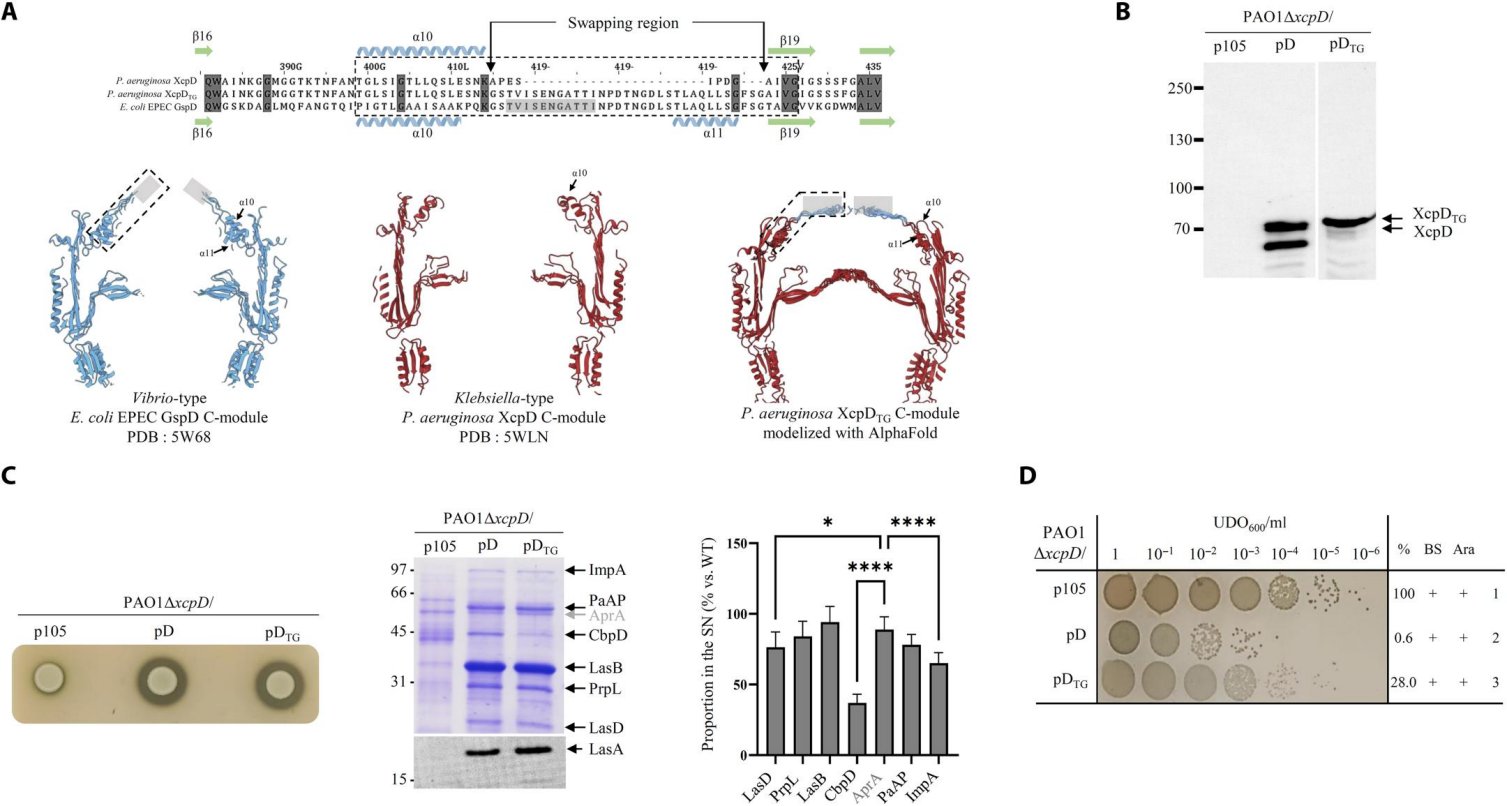
The top gate of GspD reinforces the sealing of XcpD without imposing additional selectivity. (**A**) Top gates are dispensable apical structures found in *Vibrio*-type secretins. They are formed by the convergence of 15 loops extending between α10 and α11 (see also Fig. 1C) and connected by a poorly defined region (light gray box). Top: Sequence alignment of the top gate of *Klebsiella*-type *P. aeruginosa* XcpD and *Vibrio*-type EPEC GspD. *P. aeruginosa* XcpD_TG_ is a variant with an artificially top-gated XcpD secretin. Conserved residues are highlighted in dark gray, and the corresponding secondary structures are shown. Bottom: Section view of the AlphaFold2 structural models of the corresponding GspD, XcpD, and XcpD_TG_ C-modules. (**B**) Immunoblotting analysis of OM protein samples from *P. aeruginosa* strains under denaturing conditions. The *ΔxcpD* mutant strain PAO1 was complemented by the empty vector (p105), the WT *xcpD* gene (pD), or the artificially top-gated mutant *xcpD*_TG_ (pD_TG_). (**C**) Functional investigation by in vivo plate secretion assay (left) or extracellular identification of T2SS effectors (middle). Identification of T2SS effectors was done by Coomassie blue staining or immunoblotting (LasA). Molecular mass markers (in kilodaltons) are indicated on the left. The non-T2SS–secreted effector AprA, indicated in light gray, is used as reference for T2SS-dependent secretion. Right: Quantification of the bands on the Coomassie blue staining or immunoblotting by a one-way analysis of variance (ANOVA) analysis. Only significant changes in secretion are reported (*P ≤ 0.05 and ****P ≤ 0.0001). (**D**) Bile salt sensitivity assay of *ΔxcpD* PAO1 strains producing WT or top-gated XcpD secretins performed and presented as in Fig. 3B (bottom).

The increasing number of T2SS secretin 3D structures has revealed the existence of two subclasses defined by the presence, or not, of an extra apical loop, which forms an additional apical constriction in oligomers, called top gate (Figs. 1C and 4A) (*4*). Given that the secretin XcpD belongs to the top gate–less *Klebsiella-*type subclass, we constructed a chimeric XcpD secretin harboring the top gate–corresponding region of the enteropathogenic *Escherichia coli* (EPEC) GspD (XcpD_TG_) (Fig. 4A) to assess the impact of top gate on T2SS secretion specificity and its possible role in protection against toxic foreign molecules. After producing the XcpD_TG_ chimera in the Δ*xcpD* strain by transcomplementation, we followed its fate by extraction of the OM fraction and Western blotting, under denaturing or nondenaturing conditions. This showed that the addition of a top gate into XcpD does not affect its production, OM localization, and oligomerization (Fig. 4B and figs. S1 and S2). The monomeric form, generated under denaturing conditions, logically migrates at a slightly higher molecular weight due to the presence of an extra loop (Fig. 4B).

In addition, plate secretion assay indicates that XcpD_TG_ can fully restore the T2SS-dependent extracellular protease activity in the Δ*xcpD* strain (Fig. 4C, left). A more detailed analysis of the secretion profile of the strain producing the XcpD_TG_ chimeric secretin (Fig. 4C, middle and right, and fig. S3) revealed that while most T2SS effectors are found in the culture supernatant at levels close to the WT, the secretion of CbpD, ImpA, and LasD is significantly affected compared to the control AprA. Such a phenotype demonstrates that top gate is not an additional determinant in T2SS secretion specificity (*31*) because the grafting on XcpD of a top gate belonging to a foreign secretin involved in the secretion of different and incompatible effectors still allows the secretion of Xcp effectors. However, the significant impairment of CbpD secretion (Fig. 4C and fig. S3) indicates that the top gate induces substrate-specific constraints modulating their secretion process. We are speculating that the peculiar secretion behavior of CbpD, in comparison to the other Xcp T2SS effectors, may be attributed to its nonglobular, modular organization, constituted of three modules linked by two flexible sequences (*32*). The modularity of CbpD was confirmed by our recent structural study (*28*). It can therefore be speculated that CbpD exists under different conformations, either extended or more compact. This may affect the homogeneous diffusion of this molecule, particularly when an obstacle such as the top gate is encountered. To evaluate the possible protecting role conferred by the top gate in XcpD_TG_, we tested its capacity to protect against bile salt penetration. Data presented in Fig. 4D and fig. S5F indicate that the additional top gate at the tip of XcpD significantly increased bacterial protection against bile salt under secretin overproduction. This watertightness gain of function is compatible with the apical constriction imposed by the presence of the top gate. Overall, our data show that the optional top gate apical constriction confers a better resistance to external toxic compounds but does not impose an additional specific secretion step such as those previously described between effectors and Xcp components, conferring the strict specificity of the T2SS effectors for a given nanomachine (*33*–*36*).

### Top gate identification and distribution across T2SS secretins

The gain of function brought by the presence of the optional top gate prompted us to further identify and analyze the T2SS secretins harboring this apical appendage. Multiple sequence alignments (MSAs) of a first dataset of 13 experimentally validated T2SS secretins (*37*) revealed a poor conservation of the “swapping region” used to construct the XcpD_TG_ chimera (fig. S7A). A closer analysis of the sequences indicated that the edges of the nonconserved region, bearing or not the top gate, extend well beyond the swapping region until the extensively conserved [QN]W and [AVMG]LI[VILMF] motifs at its left and right sides, respectively (fig. S7A). We extended the analysis of this large region that we called “apical region,” first, by clustering the 10,910 T2SS secretins extracted from the InterPro database. We selected the 10 largest (of 538) clusters that included 6167 sequences. In these clusters, we retrieved 32 reference sequences (including the 13 previous ones), which were previously described to have functional T2SS activities.

**Fig. 5.**
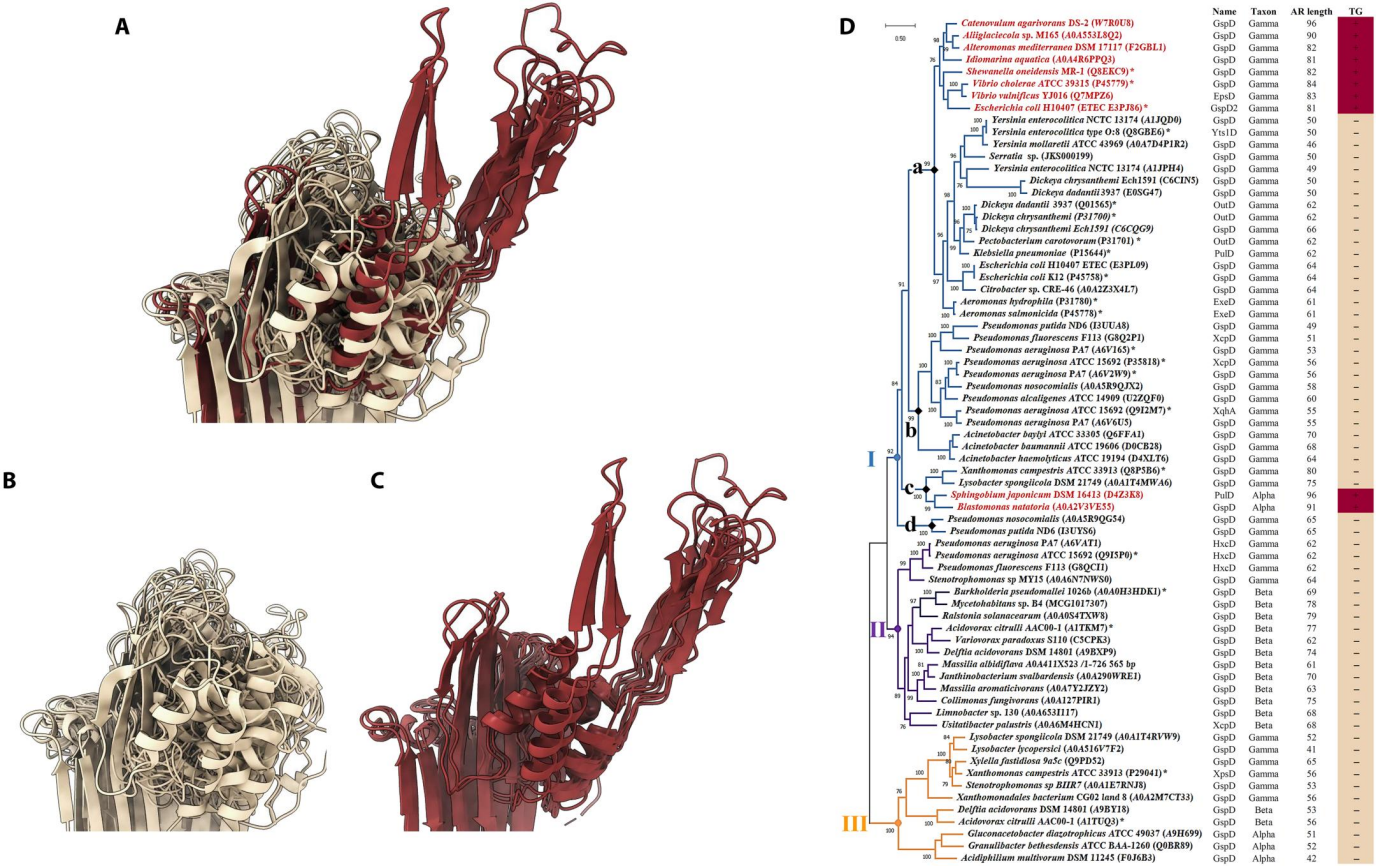
3D structure modeling and maximum likelihood phylogenetic tree inferred from 70 T2SS secretins of α/β/γ-proteobacteria. (**A**) Structural alignments of the 70 secretins showing the absence (**B**) or the presence (**C**) of the two β strands forming the top gate in the 10 secretins with an apical region’s length > 80 amino acid in size. (**D**) Phylogenetic tree displaying BP values, which were obtained following 1000 replicates and are reported next to the branch nodes. The red color of branches and taxonomic names correspond to top-gated secretins with an apical region > 80 amino acid. * denotes reference secretins with their corresponding species and strains. On the right of the tree indicated the names of the secretins and the taxonomic class of the species. The two last columns indicate the number of amino acids composing the apical region and if it contains a top gate (+) or not (−).

Next, sequence analysis confirmed the very high variability of the apical region in terms of length (from 34 to 101 amino acids) (fig. S7) and amino acid composition (from 0 to 16% for positively charged, 0 to 18 % for negatively charged, and 7 to 49% for polar residues). In contrast to their primary sequences, the 3D structures of secretin’s apical domains extremities indicated the presence or not of a top gate at the tip of both *Klebsiella* and *Vibrio* T2SS secretin types (fig. S7B). We therefore examined the 3D structure models of a representative subset of 70 selected T2SS secretin sequences constituted by an average of seven representative sequences from each of the 10 previously identified clusters (fig. S8). As revealed by the superposition of the 3D folding of the 70 corresponding apical regions, the presence of a top gate was identified in 10 of the 70 secretins (14%) (Fig. 5, A to C). Critically, these 10 top-gated secretins exhibited an apical region with a size equal to or higher than 80 residues (figs. S7 and S8). Applying this threshold to the 6167 analyzed secretins, we estimate that 25% should have a top gate, making this structural feature and the associated gain of function more common than initially expected based on the available cryo-EM 3D structures (*6*). To further explore the top gate evolution in T2SS secretins, we inferred a maximum likelihood phylogenetic tree from the 70 T2SS secretins (Fig. 5D). Overall, the evolutionary tree shows that secretins have diverged into three distinct and well-supported major clades I, II, and III [bootstrap (BP) > 90%] in α/β/γ-proteobacteria species. Clade I is the longest and the most diversified branch, including 43 secretins that diversified into four subclades (a, b, c, and d). Among them, 8 of the 10 top gate secretins are recovered in a small monophyletic sister branch inside subclade Ia, all from γ-proteobacteria (red color, apical region length > 80 amino acid; Fig. 5D). A second sister branch in subclade Ic contains the two remaining top-gated secretins (red color) both from α-proteobacteria. The remaining 60 (86% of 70 examined) secretins predicted as non–top-gated—with an apical region length < 80 amino acid—clustered into the other subclades I, the clade II, and the clade III.

Overall, our sequence analysis and phylogenetic data reveal that among proteobacteria, a substantial number of species harbors a top gate at their apical extremity, probably involved in a protective gain of function against the external environment. The presence or absence of a top gate entails two evolutionary scenarios that could have occurred during T2SS secretin evolution. First, the ancestral T2SS secretin was devoid of top gate, suggesting that acquisition of this optional gate probably conferred an additional function important for the corresponding species to adapt and/or colonize specific ecological niches. This indicates that the secretin apical region might had evolved more by diverse nucleic insertions than deletions in these species. To address a possible acquisition of top-gated secretin from one subclade to another by lateral gene transfer, we calculated and compared secretin and genome percent guanine-cytosine (GC%s) of strains belonging to the two Ia and Ic subclades (table S1 and Fig. 5D). The similar GC% recovered between secretin and corresponding genome sequences within each strain, which, however, present highly different GC% between the two subclades, rules out a possible lateral secretin gene transfer between the two subclades. This result is supported by the low identity of amino acid sequences (ranged from 30 to 34%) reported between the top-gated secretins belonging to the two subclades (tables S2, A and B). The alternative hypothesis, i.e., loss of the ancestral secretin top gate apical region, is unlikely because the non–top-gated secretins branched not only in the ancestral clade III but also in the clades I and II where they represent large fractions (86% of 70 and 75% of the 6167 examined secretins) in several and diverse lineages.

As secretins are recovered in many systems assembling large structures, it has always been of high interest to study them with the goal of understanding how these giant OM pores can transport large structure without breaching cell integrity. We hypothesized the involvement of specific gating mechanisms, which we demonstrated experimentally by performing structure-guided rCys-XL. The dynamic mechanisms of bacterial secretins presented in this study illustrate how evolution has overcome the important physical constraints imposed by the size of T2SS effectors that require intracellular folding. Thus, a subtle compromise has been reached to allow the secretion of large effectors while maintaining the integrity of the envelope. Our work paves the way to the better understanding of these fascinating molecular mechanisms found within multiple bacterial nanomachines which, unlike 3D structures, remain unpredictable.

## MATERIALS AND METHODS

### Plasmids and bacterial strains

Plasmids used in this study are listed in table S3. Polymerase chain reactions (PCRs) were performed using the Q5 high-fidelity DNA polymerase (New England BioLabs) for routine cloning or Pfu Turbo (Agilent) for quick-change mutagenesis. Custom oligonucleotides, listed in table S4, were synthesized by Integrated DNA Technologies. *P. aeruginosa* PAO1 and EPEC chromosomal DNA were used as a template for PCR to construct the chimeric version XcpD_TG_. More precisely, the regions of XcpD corresponding to residues 1 to 414 and 425 to 658 were amplified separately using primer pairs XV/XVI and XIX/XX, respectively, and the top gate region of GspD_EPEC_ corresponding to residues 417 to 449 was amplified using primer pair XVII/XVIII. The three fragments were then concatenated using primers XV and XX, and the resulting *xcpD_TG_* fragment was cloned into p105 vector using SLIC technology. The construction of Cys variants was done by quick-change mutagenesis using pD as template and the primers listed in table S4 (*21*). All constructs have been verified by DNA sequencing (Eurofins Genomics). The *E. coli* K-12 strains DH5α and TG1 were, respectively, used for cloning and site-directed mutagenesis and cultured in Luria-Broth (LB; Difco) with antibiotics as required [kanamycin (25 μg/ml) or gentamycin (20 μg/ml)]. *P. aeruginosa* PAO1 WT (laboratory collection) or Δ*xcpD* (*38*) strains were used for in vivo Cys cross-linking. The transfer of plasmids from *E. coli* to *P. aeruginosa* was performed using the helper strain 1047/pRK2013 in triparental conjugation, as described in (*39*). Transconjugants were selected on *Pseudomonas* isolation agar (Difco) supplemented with gentamycin (80 μg/ml) and cultured either in LB or tryptic soy broth (TSB, Difco) supplemented with gentamycin (50 μg/ml).

### Liquid growth conditions

*P. aeruginosa ΔxcpD* strains harboring either the empty vector p105 or the plasmid pD_TG_ or the plasmid pD encoding the different Cys variants were grown overnight at 37°C in LB medium and gentamycin. These cultures were used to inoculate 25 ml of cultures in TSB medium and gentamycin at an initial optical density at 600 nm (OD_600_) of 0.07. When OD_600_ = 0.8, filtered l-arabinose was added in the cultures at final concentration of 0.05% to induce production XcpD variants. Cells were grown for additional 3 hours, and then, the equivalents of 20 OD units were centrifuged at 2500*g* for 15 min at 4°C. The pellets were used for OM preparation.

### Preparation of IM and OM fractions

Pellets equivalent to 20 OD units were resuspended into 1 ml of buffer A [50 mM tris (pH 8), 150 mM NaCl, 10 mM EDTA, and 10 mM *N*-ethylmaleimide] supplemented with cOmplete, EDTA-free Protease Inhibitor Cocktail (Roche). The cells were disrupted by sonication (four cycles of 15 s) and then incubated on ice for 30 min. A short centrifugation at 2500*g* for 5 min at 4°C was used to pellet unbroken cells, and the supernatants were centrifugated at 125,000*g* for 45 min at 4°C. The pellets corresponding to the total membrane extracts were resuspended into 300 μl of buffer A supplemented by 2% *N*-lauroylsarcosine sodium salt (Sigma-Aldrich) and incubated on an end-to-end wheel for 30 min at room temperature (RT). Following a last centrifugation at 125,000*g* for 45 min at 4°C, the pellets corresponding to the OM fraction (*40*) were resuspended in 200 μl of 1× Laemmli buffer [62.5 mM tris-HCl (pH 6.8), 10% glycerol, 2% SDS, and 0.01% bromophenol blue] and used for SDS–polyacrylamide gel electrophoresis (SDS-PAGE) and Western blot analysis, while the supernatants corresponding to the IM fraction were mixed with one-fourth of 4× Laemmli buffer and used for SDS-PAGE and Western blot analysis.

### Plate secretion assay

*P. aeruginosa* Δ*xcpD* strains harboring either the empty vector p105 or the plasmid pD_TG_ or the plasmid pD encoding the different Cys variants were grown 7 hours at 37°C in LB medium and gentamycin. After growth, 5 μl of cultures was spotted on TSA (Difco)–skim milk (Difco) plates supplemented with gentamycin (50 μg/ml) and, when indicated, with a specific concentration of DTT depending on the variants: 5 mM for V125C and 7 mM for T477C and S480C. The plates were incubated for 16 hours at 37°C to allow the formation of the degradation halos around colonies. For all variants presenting a phenotype, we checked that the amino acid substitutions did not produce substantial growth alteration (fig. S9).

### Bile salt sensitivity assay on plate

*P. aeruginosa ΔxcpD* strains harboring either the empty vector p105 or the plasmid pD_TG_ or the plasmid pD encoding the different Cys variants were grown 7 hours at 37°C in LB medium supplemented with gentamycin (50 μg/ml). After growth, the cultures were first diluted to an OD_600_ = 1 and then serially 10-fold diluted in 96-well plates. Last, 4 μl of each dilution was spotted on LB agar plate without NaCl containing gentamycin (50 μg/ml) also supplemented, when indicated, with 0.05% arabinose, 2% bile salt (Sigma-Aldrich), and/or 1 mM DTT for V125C and/or 3 mM DTT for T477C and S480C. Experiments were performed in biological triplicates. Colony-forming units were counted for all strains and expressed as percentage compared to the reference strain PAO1Δ*xcpD*/p105. Significant differences between strains were calculated by a one-way analysis of variance (ANOVA) test with Tukey’s correction using GraphPad Prism software.

### Preparation of extracellular proteins

The supernatants of cell cultures were precipitated by trichloroacetic acid (TCA) precipitation technique. TCA was added into culture supernatants at 12% final concentration and incubated overnight at 4°C. After centrifugation at 16,000*g* for 30 min at 4°C, the pellets were washed two times with 1 ml of 90% cold acetone. Last, the pellets were dried for 5 min at RT and resuspended in Laemmli buffer for further analysis by SDS-PAGE. Identities of the *P. aeruginosa* secreted proteins annotated in Fig. 4C have previously been confirmed by mass spectrometry (*41*, *42*).

### SDS-PAGE and Western blot analysis

For analysis of protein profiles under denaturing conditions, IM, OM, and supernatant protein samples were heated for 10 min at 95°C and separated on 12% acrylamide SDS-PAGE. As defined in figures, protein samples were analyzed under oxidative conditions (samples suspended in 1× Laemmli buffer) or reducing conditions (samples suspended in 1× Laemmli buffer +5% (v/v) βME). For analysis of secretin oligomers, the corresponding OM protein samples were treated with 4 M urea in 1× XT-tricine buffer (Bio-Rad) for 1 hour at RT to solubilize aggregates and incubated for 15 min at 45° or 95°C to maintain or not secretin oligomers. Both heat-denaturated and non–heat-denaturated protein samples were analyzed on a 3 to 8% tris-acetate acrylamide precast gel (Criterion, Bio-Rad). After separation, the proteins were either stained with Coomassie blue (10% acetic acid, 50% ethanol, and 0.125% Coomassie blue R-250) or transferred onto nitrocellulose membrane for Western blot analysis. Transfers were performed with semi-dry technic using Power Blotter Station (Invitrogen). The membrane was washed briefly in tris-buffered saline with 0.05% Tween 20 (TBS-T) and then blocked in TBS-T + 5% milk for 1 hour at RT. Then, after three washes in TBS-T, the membrane is incubated for 1 hour at RT in TBS-T + 5% milk containing the primary antibody directed against LasA (1:2000), XcpL (1:1000), and DsbA (1:5000) for the fractionation control or against the V5-tag (1:5000) or XcpD-Nter (1:3000) for XcpD monomers, dimers, and oligomers. This was followed by five washes in TBS-T before an incubation of 1 hour at RT in TBS-T + 5% milk containing the secondary antibody anti-rabbit horseradish peroxidase–conjugated (1:5000). Last, after five more washes with TBS-T, the peroxidase reaction was revealed by chemiluminescence (SuperSignal West Dura Extended Duration Substrate, Thermo Fisher Scientific) and scanned with ImageQuant LAS 4000.

### Quantification of supernatant content

Supernatant were prepared twice in quadruplicate, analyzed by SDS-PAGE, and stained by Coomassie blue. The intensity of the bands corresponding to the T2SS effectors (ImpA, PaaP, CbpD, LasB, PrpL, and LasD) and the T1SS protein AprA were quantified using band-it tool (tools.thecodingbiologist.com). The intensities found for Δ*xcpD*/pD_TG_ were expressed relatively to the intensities for Δ*xcpD*/pD. To extract significative differences between T2SS secretion and T1SS secretion, we performed a one-way ANOVA test with Tukey’s correction using GraphPad Prism software.

### Calculation of strain generation times

*P. aeruginosa ΔxcpD* strains harboring either the empty vector p105 or plasmids encoding WT or the different XcpD secretin variants were grown overnight at 37°C in LB medium supplemented with gentamycin (50 μg/ml). These cultures were used to inoculate 100 μl of fresh LB medium with gentamycin (50 μg/ml) and 2 mM DTT, when indicated, at an initial OD_600_ of 0.06 in 96-well plates. Bacterial strains were grown 24 hours at 37°C under agitation in a TECAN microplate reader. The generation time was calculated for each strain during the exponential phase, and the data of three independent replicates were analyzed with GraphPad Prism software.

### Genomic analysis of the top gate domain in T2SS secretins

First, an MSA was performed on a dataset containing 13 secretins known to be involved in functional T2SSs (*37*) using JalView tool (*43*). To extend the analysis, a dataset of 10,910 T2SS GspD secretin sequences from the InterPro database (IPR013356) (*44*) was downloaded, and an all-versus-all sequence similarity searching and clustering (identity ≥ 50% and bidirectional coverage ≥ 70%) of this large-scale dataset was carried out using MMseqs2 algorithm (*45*, *46*). The 10 largest clusters gathering in 6167 sequences were selected, and the GspD reference secretin (UniProtKB P45779) from *V. cholerae* serotype O1 was included. Sequences of each cluster were then subjected to MSA, and their apical region was extracted and analyzed. The distribution of the apical region’s length across the 6167 secretins was summarized into a violin plot using GraphPad Prism software. Last, on the basis of the variabilities observed in the apical regions in terms of length, referenced secretins, diversity of bacterial lineages, and either single or duplicates of secretins in some strains, a dataset of 70 secretins was selected to (i) analyze the structural organization of their corresponding apical region from their 3D cryo-EM structure or structure predictions and (ii) establish possible evolutionary relationships between the phylogeny of T2SS secretins and the diversity of the top gate regions. MSA of the secretins was carried out, and last, an evolutionary tree using the maximum likelihood method under the --model LG+F+IC using raxmlHPC-PTHREADS was constructed. The nodes robustness was estimated through BP analysis of 1000 replicates.

### Structure prediction and secondary structure assignment of top gate in secretins

The model of XcpD_TG_ monomer was generated using AlphaFold2 code source (*47*), and the oligomeric state was generated by structural alignment of 15 monomers of XcpD_TG_ onto each of the 15 monomers of XcpD cryo-EM structure (Protein Data Bank: 5WLN) using Pymol v2.5 software. Images of structural model were generated using the UCSF Chimera 1.3 software. The 3D structure of secretins used in the genomic analysis originates from cryo-EM structures when possible; otherwise, we retrieved the AlphaFold predictions from the AlphaFold Protein Structure Database (*48*). Last, we searched manually for the presence of the strands β17 and β18 forming the top gate on the structure using Pymol and reported their presence or not in the phylogenetic tree.

## Supplementary Materials

### This PDF file includes:

Figs. S1 to S9 Tables S1 to S4 References
