# Supplementary material for: Insights into dynamics and gating properties of T2SS secretins
Source: Sci Adv. 2023 Oct 4;9(40):eadg6996. doi: 10.1126/sciadv.adg6996 (PMC10550240; doi:10.1126/sciadv.adg6996)
Supplement: Supplementary file 1 — Figs. S1 to S9 Tables S1 to S4 References [file sciadv.adg6996_sm.pdf]

Science Advances
AAAS

Supplementary Materials for

Insights into dynamics and gating properties of T2SS secretins

Brice Barbat *et al.*

Corresponding author: Romé Voulhoux, voulhoux@imm.cnrs.fr

Sci. Adv. **9**, eadg6996 (2023)
DOI: 10.1126/sciadv.adg6996

This PDF file includes:

Figs. S1 to S9
Tables S1 to S4
References

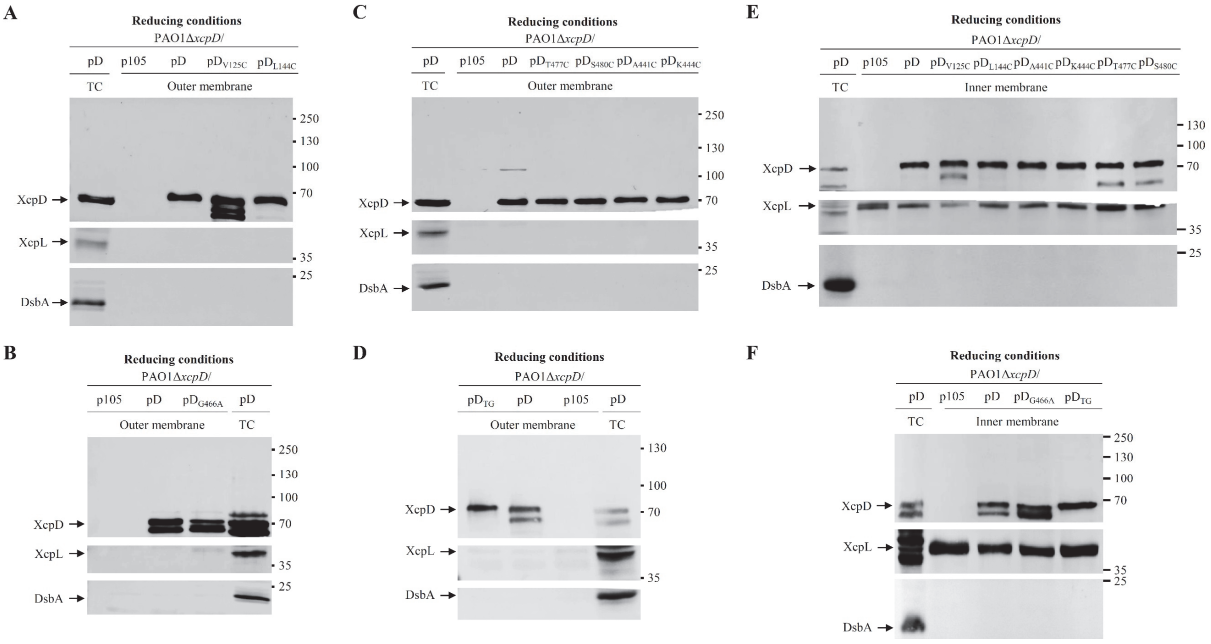

Supplementary Figure S1. Fractionation control for XcpD's cysteine variants. Immunoblotting of outer and inner membrane protein samples under βME-induced reducing conditions using anti-V5 (panels A, C and E) or anti-XcpD Nter (panels B, D and F) for secretins, anti-XcpL (IM marker) and anti-DsbA (periplasmic marker). A total cell extract (TC) has been used as a control. Molecular mass markers (in kDa) are indicated on the right.

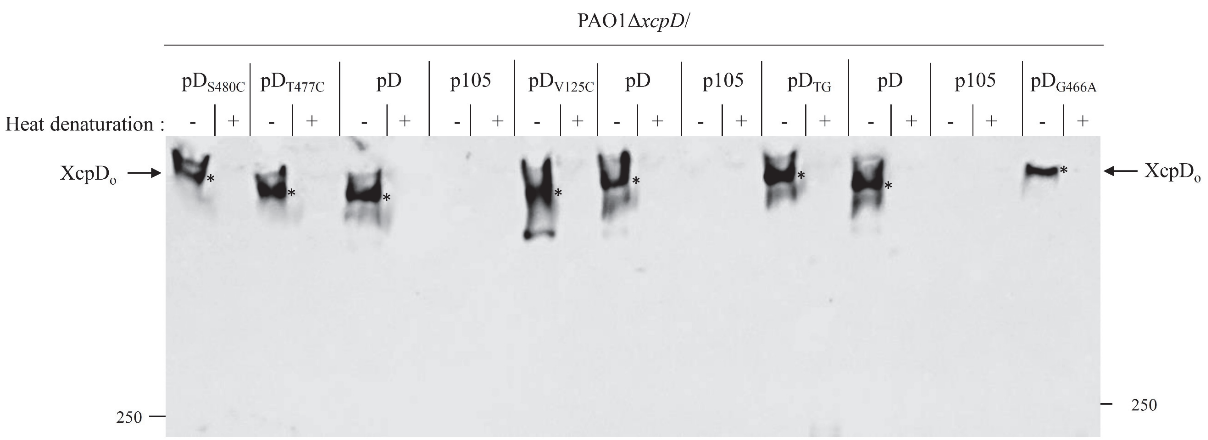

Supplementary Figure S2. Oligomerization properties of the XcpD secretin variants. The OM protein samples of PAO1 *ΔxcpD* strains complemented by the empty vector (p105), the WT *xcpD* gene (pD), or the mutant *xcpD$_{T477C}$* (pD$_{T477C}$), *xcpD$_{S480C}$* (pD$_{S480C}$), *xcpD$_{V125C}$* (pD$_{V125C}$), *xcpD$_{TG}$* (pD$_{TG}$) and *xcpD$_{G466A}$* (pD$_{G466A}$) genes were analyzed under 4M urea with (+) or without (-) heat denaturation at 95°C. The proteins were separated on a 3-8% Tris-acetate acrylamide gradient precast gel and further analyzed by immunoblotting using an anti-XcpD-Nter to specifically reveal the urea resistant and heat sensitive secretin oligomers (XcpD$_{o}$ (*)). The 250 kDa molecular mass marker is indicated on left and right sides.

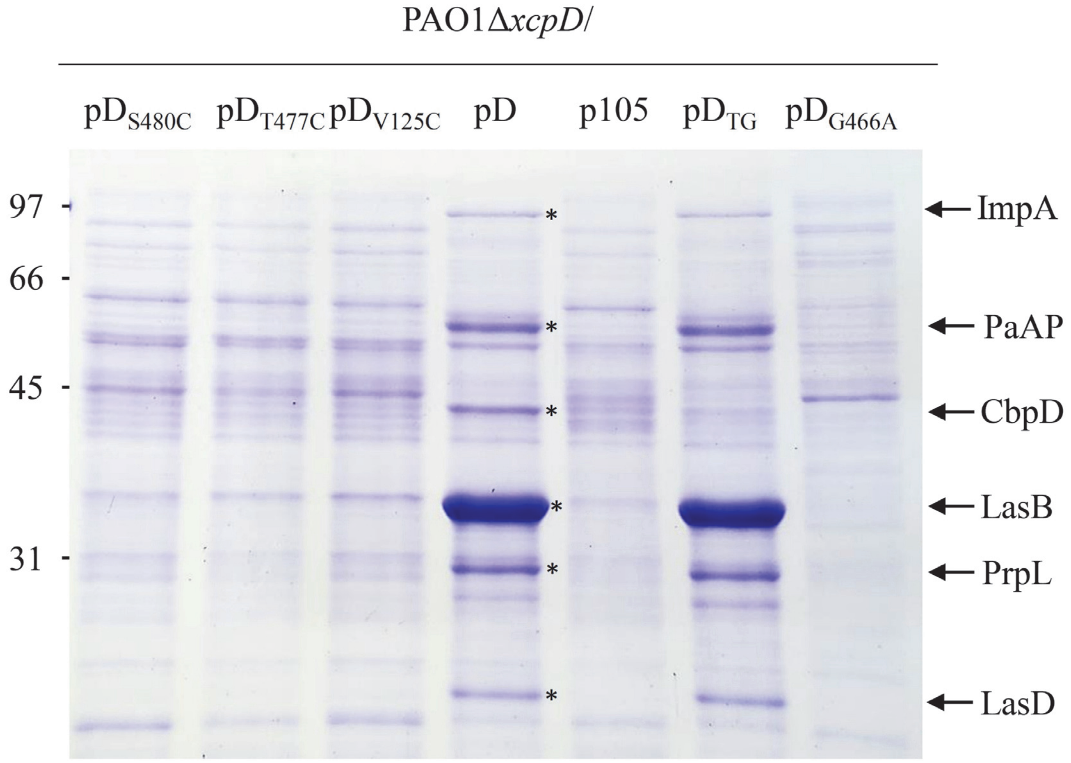

Supplementary Figure S3. Xcp T2SS secretion profile of the XcpD secretin variants. Extracellular detection of the six major Xcp T2SS effectors by SDS-PAGE Coomassie blue staining of bacterial supernatant protein samples. The six Xcp T2SS effectors investigated are indicated by an asterisk (*) in the WT XcpD positive control and by their name on the right. Molecular mass markers (in kDa) are indicated on the left.

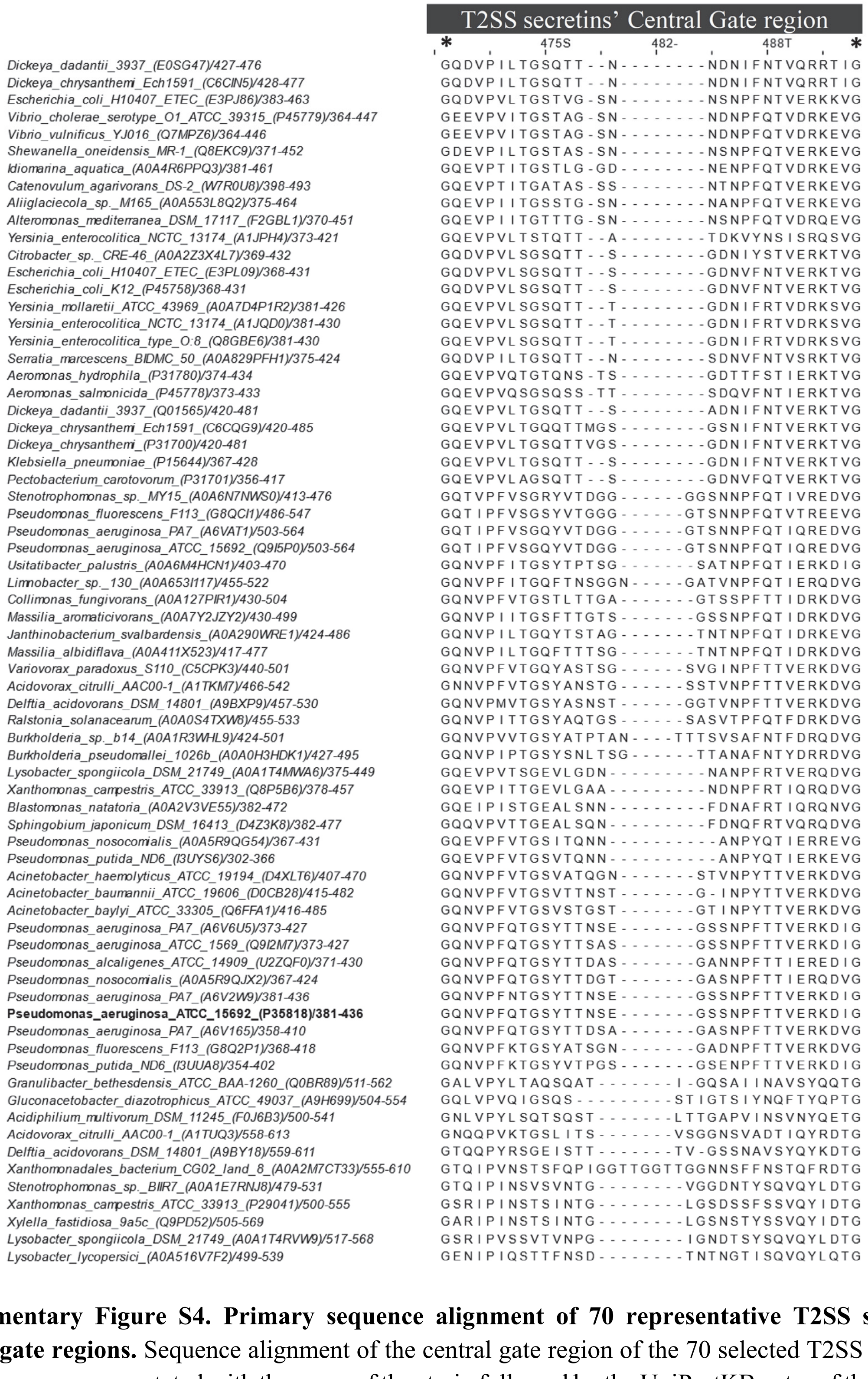

Supplementary Figure S4. Primary sequence alignment of 70 representative T2SS secretins' central gate regions. Sequence alignment of the central gate region of the 70 selected T2SS secretins. The sequences are annotated with the name of the strain followed by the UniProtKB entry of the secretin. The strictly conserved glycine pivots are indicated by an asterisk (*). The numbers indicated at the top are the amino acids counting using the sequence of *Pa*XcpD as a reference (bold).

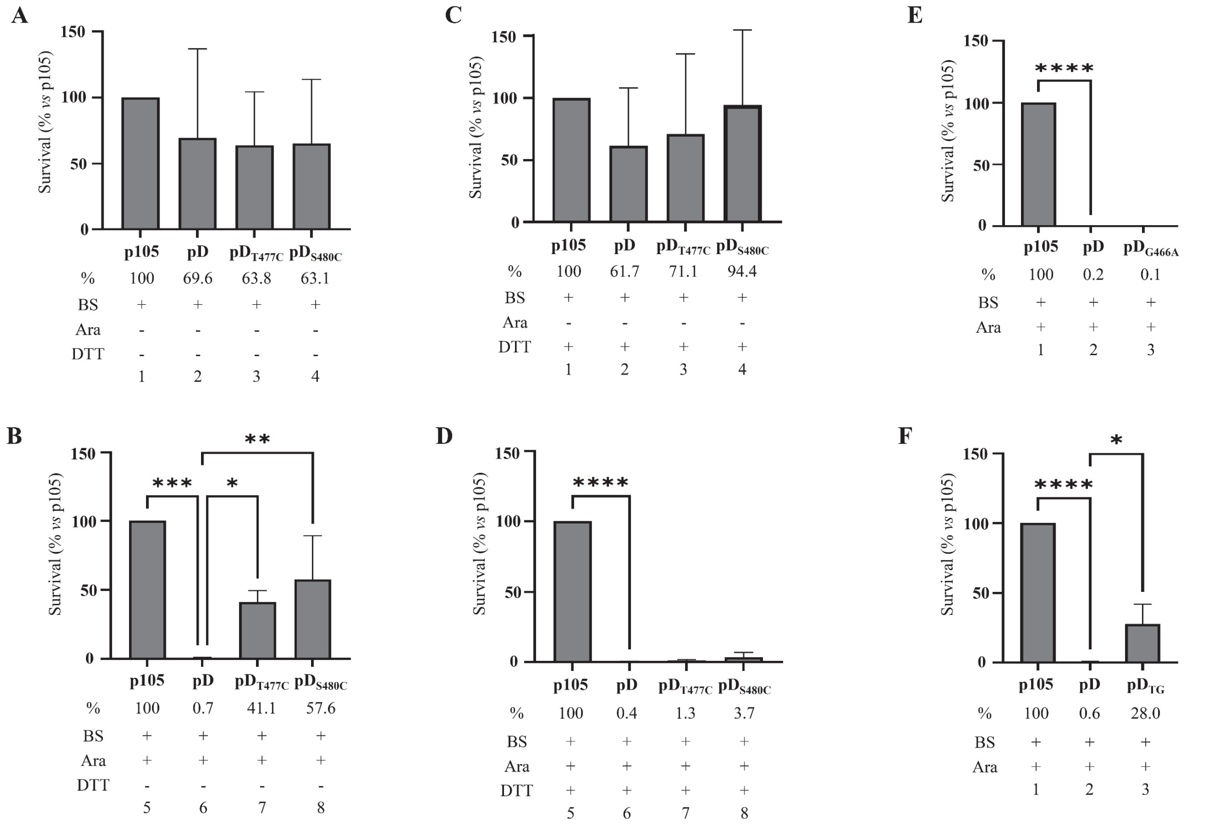

Supplementary Figure S5. Bile salt sensitivity assays quantification. Bile salt sensitivity assays presented figures 3B, 3C and 4D were performed in biological triplicate. Colony forming units were counted and presented as a mean percentage relative to the reference strain PAO1Δ*xcpD*/p105. A one-way ANOVA analysis was performed as statistical. Only significant changes in bile salt sensitivities are reported (* for P ≤ 0.05, ** for P ≤ 0.01, *** for P ≤ 0.001 and **** for P ≤ 0.0001). Data are presented following the nomenclature used figures 3 and 4.

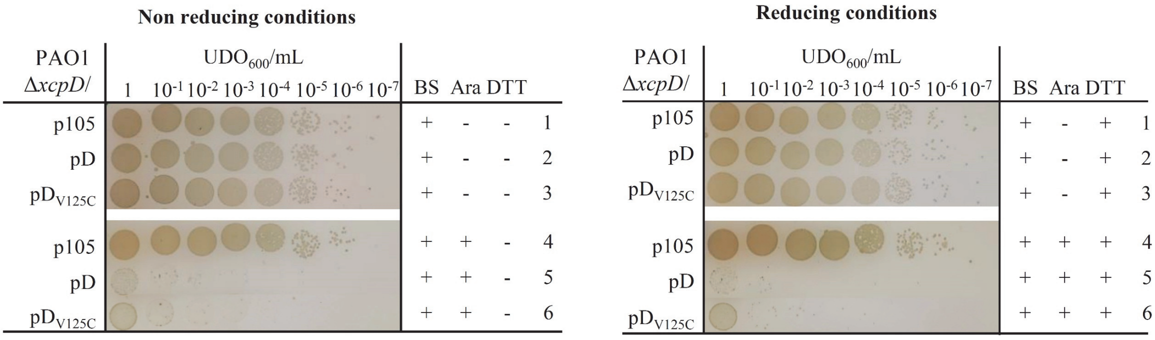

Supplementary Figure S6. Bile salt sensitivity assay of the XcpD$_{V125C}$ secretin variant. Bile salt sensitivity assay on plate under non-reducing or DTT-induced reducing conditions. Growth of the different strains was tested after spotting serial 10-fold-dilutions on 2 % bile salt (BS) agar plates, supplemented (+) or not (-) with 0.05 % arabinose (Ara) and/or 1 mM DTT.

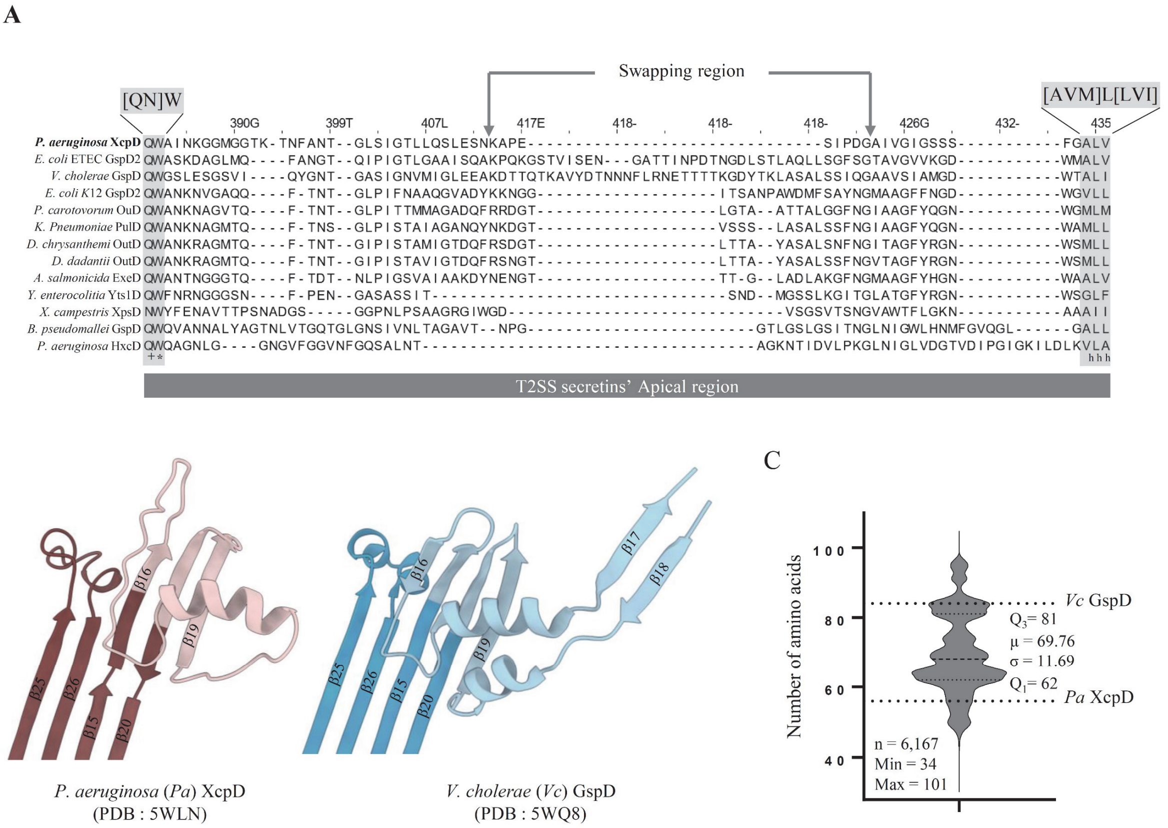

Supplementary Figure S7. Primary and ternary structural organization of apical regions of T2SS secretins. (**A**) Sequence alignment of 13 known secretins from functional T2SS (*42*) shows that the apical region (amino acids 380-436 in XcpD) is delimitated by two well conserved patches of residues highlighted in grey ([QN]W and [AVM]L[LVI]), which comprise the top gate sequence. The numbers indicated at the top are the amino acids counting using the sequence of XcpD as a reference (bold). (**B**) 3D structure prediction of *Pa*XcpD (red) and *Vc*GspD (blue) where the apical regions, based on sequence alignments, have been coloured in light red and light blue, respectively. (**C**) Violin plot representing the distribution of the apical region's (AR) length for the 6,167 secretins analyzed using GraphPad – Prism software.

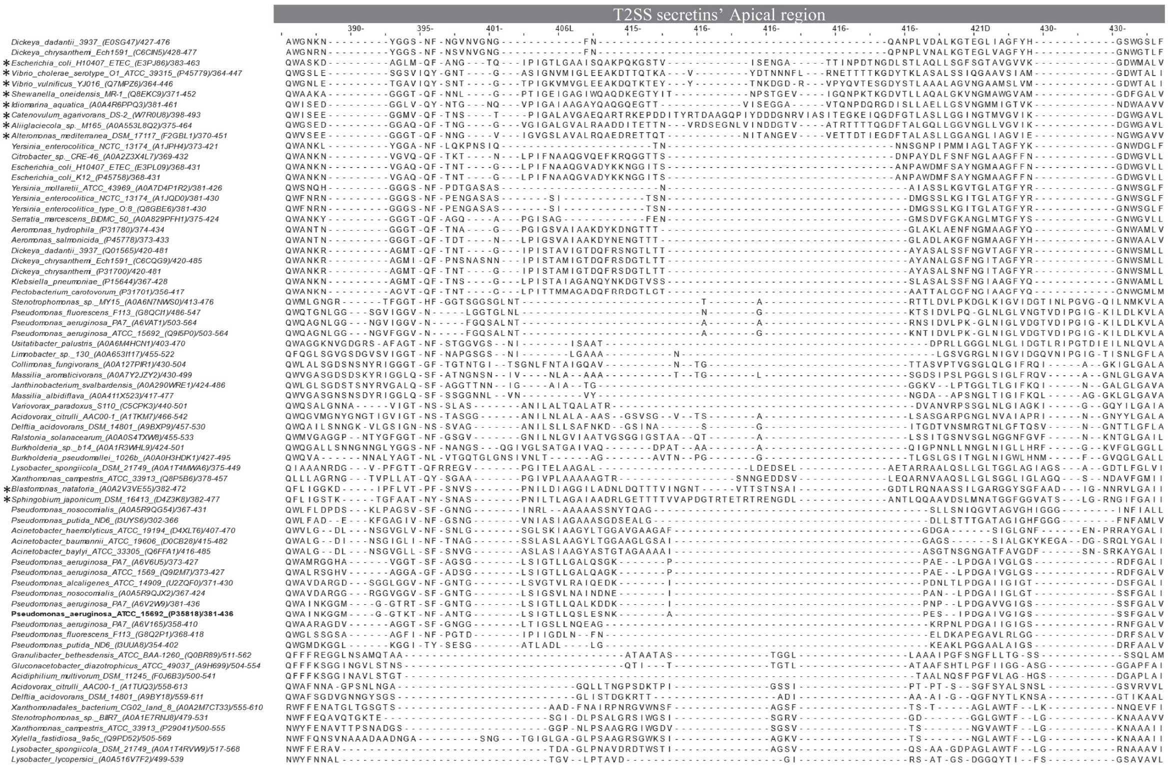

Supplementary Figure S8. Genomic analysis of apical regions of T2SS secretins. Sequence alignment of the apical region of the 70 selected T2SS secretins. The sequences are annotated with the name of the strain followed by the UniProtKB entry of the secretin. The numbers indicated at the top are the amino acids counting using the sequence of *P. aeruginosa* XcpD as a reference (bold). The 10 top-gated secretins are indicated by an asterisk (*).

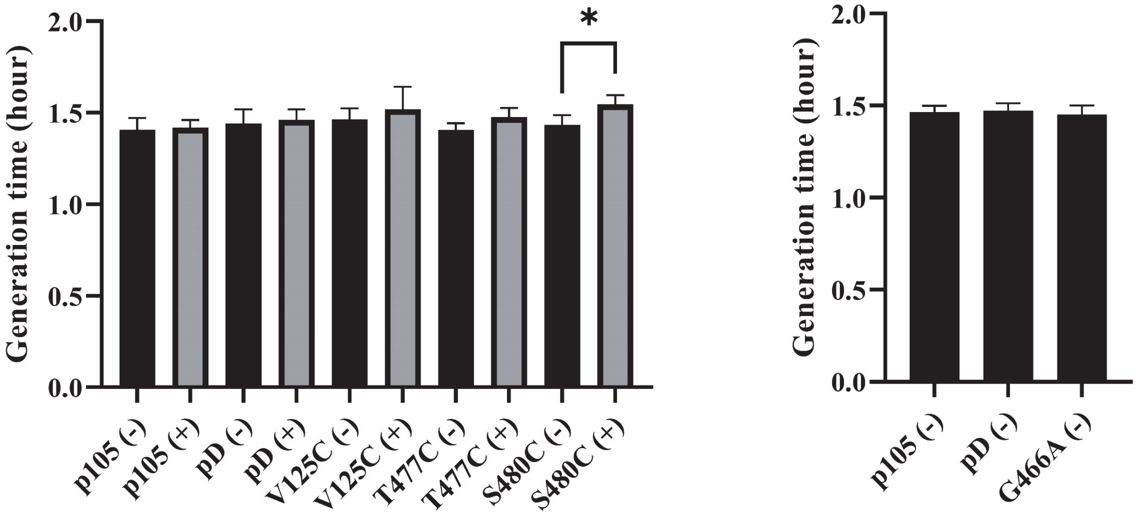

Supplementary Figure S9. Growth comparison between strains expressing the secretin variants under oxydative or reductive conditions. Each PAO1 *ΔxcpD* strains complemented by the empty vector (p105), the WT *xcpD* gene (pD), or the mutant $xcpD_{V125C}$ (V125C), $xcpD_{T477C}$ (T477C), $xcpD_{S480C}$ (S480C), $xcpD_{G466A}$ (G466A) have been cultivated without (-) or with 2 mM DTT (+) and the generation time during exponential growth has been extracted. A biological triplicate has been done and a one-way ANOVA analysis with Tukey's correction has been performed as statistical analysis (*p < 0.05 or not signifcant in all other cases).

Supplementary Table S1: GC% of the genomes and secretins' genes of four proteobacteria found in the red clusters of the phylogenetic tree (Fig. 5D).

**Strain description**	**Genome GC%**	**T2SS secretin* GC%**
1. Blastomonas natatoria DSM 3183	63.40	65.0
2. Sphingobium japonicum DSM 16413	64.88	67.2
3. Vibrio cholerae serotype O1 ATCC 39315/N16961	47.49	49.8
4. Escherichia coli H10407 ETEC	50.73	54.8

* Genomic coordinates of secretins: 1. NZ_QJJM01000001.1:108821-111019, 2. NC_014006.1:c2370518-2368338, 3. NZ_LT906614.1:2905211-2907232 and 4. FN649414.1:3493773-3495830

Supplementary Table S2A. Best blast hits of the *Sphingobium japonicum* DSM 16413 secretin *vs* the closest secretins from the phylogenetic tree (Fig. 5D).

Description	Max Score	Total Score	E value	Per. ident	Acc. Len
Sphingobium japonicum DSM 16413 (D4Z3K8)	1437	1437	0.0	100	726
Blastomonas natatoria DSM 3183 (A0A2V3VE55)	779	779	0.0	60.09	732
Xanthomonas campestris ATCC 33913 (Q8P5B6)	561	561	0.0	49.33	690
Lysobacter spongiicola DSM 21749 (A0A1T4MWA6)	536	536	0.0	48.45	674
Alteromonas mediterranea DSM 17117 (F2GBL1)	361	361	3E-117	35.12	681
Stenotrophomonas sp. MY15 (A0A6N7NWS0)	358	358	7E-115	35.25	793
Escherichia coli H10407 ETEC (E3PJ86)	343	343	3E-110	34.59	686
Pseudomonas nosocomialis (A0A5R9QG54)	329	329	2E-105	35.26	643
Aliiglaciecola sp. M165 (A0A553L8Q2)	330	330	3E-105	34.55	692
Usitatibacter palustris (A0A6M4HCN1)	326	326	1E-103	34.40	689
Idiomarina aquatica (A0A4R6PPQ3)	320	320	1E-101	32.30	693
Acinetobacter baylyi ATCC 33305 (Q6FFA1)	321	321	4E-101	32.47	772
Delftia acidovorans DSM 14801 (A9BXP9)	320	320	1E-100	34.36	787
Acinetobacter baumannii ATCC 19606 (D0CB28)	312	312	8E-98	31.53	756
Vibrio vulnificus YJ016 (Q7MPZ6)	309	309	1E-97	32.67	672
Acinetobacter haemolyticus ATCC 19194 (D4XLT6)	310	310	4E-97	32.89	735
Burkholderia pseudomallei 1026b (A0A0H3HDK1)	307	307	6E-96	33.67	757
Pseudomonas aeruginosa ATCC 15692 (P35818)	303	303	3E-95	33.33	658
Pseudomonas aeruginosa PA7 (A6V2W9)	301	301	7E-95	33.08	658
Catenovulum agarivorans DS-2 (W7R0U8)	299	299	3E-93	32.35	730
Janthinobacterium svalbardensis (A0A290WRE1)	298	298	6E-93	32.37	745
Variovorax paradoxus S110 (C5CPK3)	296	296	7E-92	33.80	774
Pseudomonas nosocomialis (A0A5R9QJX2)	290	290	9E-91	32.54	645
Pseudomonas aeruginosa PA7 (A6V165)	286	286	3E-89	33.58	634
Massilia albidiflava (A0A411X523)	287	287	8E-89	32.95	726
Vibrio cholerae serotype O1 ATCC 39315 (P45779)	285	285	1E-88	32.99	674
Shewanella oneidensis MR-1 (Q8EKC9)	285	285	3E-88	32.20	704
Pseudomonas aeruginosa ATCC 1569 (Q9I2M7)	283	283	7E-87	32.26	776
Pseudomonas fluorescens F113 (G8QCI1)	281	281	5E-86	31.16	786

Supplementary Table S2B. Best blast hits of the *Blastomonas natatoria DSM 3183* secretin *vs* the closest secretins from the phylogenetic tree (Fig. 5D).

Description	Max Score	Total Score	E value	Per. ident	Acc. Len
Blastomonas natatoria DSM 3183 (A0A2V3VE55)	1446	1446	0.0	100.0	732
Sphingobium japonicum DSM 16413 (D4Z3K8)	769	769	0.0	58.05	726
Xanthomonas campestris ATCC 33913 (Q8P5B6)	509	509	3E-174	45.36	690
Lysobacter spongiicola DSM 21749 (A0A1T4MWA6)	503	503	3E-172	46.66	674
Alteromonas mediterranea DSM 17117 (F2GBL1)	333	333	2E-106	34.74	681
Escherichia coli H10407 ETEC (E3PJ86)	326	326	6E-104	34.65	686
Delftia acidovorans DSM 14801 (A9BXP9)	317	317	3E-99	33.20	787
Ralstonia solanacearum (A0A0S4TXW8)	314	314	3E-98	34.91	806
Usitatibacter palustris (A0A6M4HCN1)	311	311	3E-98	34.78	689
Pseudomonas nosocomialis (A0A5R9QG54)	310	310	4E-98	35.22	643
Aliiglaciecola sp. M165 (A0A553L8Q2)	309	309	3E-97	33.83	692
Stenotrophomonas sp. MY15 (A0A6N7NWS0)	305	305	6E-95	36.32	793
Collimonas fungivorans (A0A127PIR1)	301	301	2E-93	32.91	760
Shewanella oneidensis MR-1 (Q8EKC9)	295	295	9E-92	32.99	704
Idiomarina aquatica (A0A4R6PPQ3)	291	291	1E-90	32.65	693
Vibrio vulnificus YJ016 (Q7MPZ6)	287	287	2E-89	31.35	672
Janthinobacterium svalbardensis (A0A290WRE1)	288	288	4E-89	32.17	745
Catenovulum agarivorans DS-2 (W7R0U8)	288	288	6E-89	32.04	730
Pseudomonas nosocomialis (A0A5R9QJX2)	281	281	4E-87	34.03	645
Pseudomonas aeruginosa PA7 (A6V2W9)	280	280	6E-87	32.42	658
Acinetobacter baumannii ATCC 19606 (D0CB28)	282	282	1E-86	30.11	756
Variovorax paradoxus S110 (C5CPK3)	282	282	2E-86	33.33	774
Vibrio cholerae serotype O1 ATCC 39315 (P45779)	280	280	2E-86	31.91	674
Massilia albidiflava (A0A411X523)	280	280	4E-86	32.18	726
Pseudomonas aeruginosa ATCC 15692 (P35818)	277	277	9E-86	32.06	658
Acinetobacter haemolyticus ATCC 19194 (D4XLT6)	279	279	1E-85	30.24	735
Acinetobacter baylyi ATCC 33305 (Q6FFA1)	278	278	7E-85	29.60	772
Escherichia coli K12 (P45758)	269	269	1E-82	32.57	650
Escherichia coli H10407 ETEC (E3PL09)	269	269	1E-82	32.57	650

Supplementary Table S3. Plasmids used in this study.

**Name**	**Description**	**Reference**
pRK2013	Km^R, ColE1, Tra+ Mob+ (RK2)	(*49*)
p105	Gm^R, *araC*-pBAD, pJN105 broad-host-range vector	(*50*)
pD	*xcpD* gene fragment with a C-terminal V5 tag cloned into p105 (pJN-XcpD-V5)	(*21*)
pD_{V125C}	*xcpD-V125C* gene fragment with a C-terminal V5 tag cloned into p105	This study
pD_{L144C}	*xcpD-L144C* gene fragment with a C-terminal V5 tag cloned into p105	This study
pD_{A441C}	*xcpD-A441C* gene fragment with a C-terminal V5 tag cloned into p105	This study
pD_{K444C}	*xcpD-K444C* gene fragment with a C-terminal V5 tag cloned into p105	This study
pD_{T477C}	*xcpD-T477C* gene fragment with a C-terminal V5 tag cloned into p105	This study
pD_{S480C}	*xcpD-S480C* gene fragment with a C-terminal V5 tag cloned into p105	This study
pD_{G466A}	*xcpD-G466A* gene fragment with a C-terminal V5 tag cloned into p105	This study
pD_{TG}	*xcpD* gene with *E. coli* top gate and a C-terminal V5 tag cloned into p105	This study

Supplementary table S4. Oligonucleotides used in this study.

**Primer name**	**Primer sequence**
I : *xcpD*-V125C-F<br>II : *xcpD*-V125C-R	5’-cgaccaggcgcgcatctgccccaacgccgaggcc-3’<br>5’-ggcctcggcgttggggcagatgcgcgcctggtcg-3’
III : *xcpD*-L144C-F<br>IV : *xcpD*-L144C-R	5’-gagcgcgccggatcgctgcgagacgcgggtgatc-3’<br>5’-gatcacccgcgtctcgcagcgatccggcgcgctc-3’
V : *xcpD*-A441C-F<br>VI : *xcpD*-A441C-R	5’-ctggtcaccgcgctctcgtgcaacaccaagagcaac-3’<br>5’-gttgctcttggtgttgcacgagagcgcggtgaccag-3’
VII : *xcpD*-K444C-F<br>VIII : *xcpD*-K444C-R	5’-gcgctctcggccaacacctgcagcaacctgctgtcg-3’<br>5’-cgacagcaggttgctgcaggtgttggccgagagcgc-3’
IX : *xcpD*-T477C-F<br>X : *xcpD*-T477C-R	5’-gaccggctcctgcaccaacagcgaag-3’<br>5’-cttcgctgttggtgcagtaggagccggtc-3’
XI : *xcpD*-S480C-F<br>XII : *xcpD*-S480C-R	5’-ggctcctacaccaactgcgaaggtagcaacc-3’<br>5’-cgttgcaaccttcgcagttggtggtgtaggagcc-3’
XIII : *xcpD*-G466A-F<br>XIV : *xcpD*-G466A-R	5’-gagattctggtcgcccagaacgtaccg-3’<br>5’-cggtacgttctgggcgaccagaatctc-3’
XV : *xcpD*-Nter-F<br>XVI : *xcpD*-Nter-R	5’-gaattcctgcagcccgggatcgcagaacccgccga-3’<br>5’-gatcaccgtcgagcccttgttgctctcgag-3’
XVII : *gspD*-TG-F<br>XVIII : *gspD*-TG-R	5’-ctcgagagcaacaagggctcgacggtgatc-3’<br>5’-gccgacgatggcgccaccgctaaaggaaagaagctgggc-3’
XIX : *xcpD*-Cter-F<br>XX : *xcpD*-Cter-R	5’-gcccagcttctttcctttagcggtggcgccatcgtcggc-3’<br>5’-agaactagtggatcccccgggttacgtagaatcgagaccgaggagagggttagggataggctta ccttccgtcatcagttcgcg-3’

REFERENCES AND NOTES

1. R. Conners, M. McLaren, U. Łapińska, K. Sanders, M. R. L. Stone, M. A. T. Blaskovich, S. Pagliara, B. Daum, J. Rakonjac, V. A. M. Gold, CryoEM structure of the outer membrane secretin channel pIV from the f1 filamentous bacteriophage. *Nat. Commun.* **12**, 6316 (2021).

2. M. McCallum, S. Tammam, J. L. Rubinstein, L. L. Burrows, P. L. Howell, CryoEM map of *Pseudomonas aeruginosa* PilQ enables structural characterization of TsaP. *Structure* **29**, 457–466.e4 (2021).

3. L. J. Worrall, C. Hong, M. Vuckovic, W. Deng, J. R. C. Bergeron, D. D. Majewski, R. K. Huang, T. Spreter, B. B. Finlay, Z. Yu, N. C. J. Strynadka, Near-atomic-resolution cryo-EM analysis of the *Salmonella* T3S injectisome basal body. *Nature* **540**, 597–601 (2016).

4. Z. Yan, M. Yin, D. Xu, Y. Zhu, X. Li, Structural insights into the secretin translocation channel in the type II secretion system. *Nat. Struct. Mol. Biol.* **24**, 177–183 (2017).

5. K. V. Korotkov, T. Gonen, W. G. Hol, Secretins: Dynamic channels for protein transport across membranes. *Trends Biochem. Sci.* **36**, 433–443 (2011).

6. B. Barbat, B. Douzi, R. Voulhoux, Structural lessons on bacterial secretins. *Biochimie* **205**, 110–116 (2023).

7. T. Spreter, C. K. Yip, S. Sanowar, I. André, T. G. Kimbrough, M. Vuckovic, R. A. Pfuetzner, W. Deng, A. C. Yu, B. B. Finlay, D. Baker, S. I. Miller, N. C. J. Strynadka, A conserved structural motif mediates formation of the periplasmic rings in the type III secretion system. *Nat. Struct. Mol. Biol.* **16**, 468–476 (2009).

8. A. A. Chernyatina, H. H. Low, Core architecture of a bacterial type II secretion system. *Nat. Commun.* **10**, 5437 (2019).

9. I. D. Hay, M. J. Belousoff, R. A. Dunstan, R. S. Bamert, T. Lithgow, Structure and membrane topography of the *Vibrio*-type secretin complex from the type 2 secretion system of enteropathogenic *Escherichia coli*. *J. Bacteriol.* **200**, e00521-17 (2018).

10. I. D. Hay, M. J. Belousoff, T. Lithgow, Structural basis of type 2 secretion system engagement between the inner and outer bacterial membranes. *MBio* **8**, e01344-17 (2017).

11. S. P. Howard, L. F. Estrozi, Q. Bertrand, C. Contreras-Martel, T. Strozen, V. Job, A. Martins, D. Fenel, G. Schoehn, A. Dessen, Structure and assembly of pilotin-dependent and -independent secretins of the type II secretion system. *PLOS Pathog.* **15**, e1007731 (2019).

12. J. Hu, L. J. Worrall, C. Hong, M. Vuckovic, C. E. Atkinson, N. Caveney, Z. Yu, N. C. J. Strynadka, Cryo-EM analysis of the T3S injectisome reveals the structure of the needle and open secretin. *Nat. Commun.* **9**, 3840 (2018).

13. J. Hu, L. J. Worrall, M. Vuckovic, C. Hong, W. Deng, C. E. Atkinson, B. Brett Finlay, Z. Yu, N. C. J. Strynadka, T3S injectisome needle complex structures in four distinct states reveal the basis of membrane coupling and assembly. *Nat. Microbiol.* **4**, 2010–2019 (2019).

14. M. Lunelli, A. Kamprad, J. Bürger, T. Mielke, C. M. T. Spahn, M. Kolbe, Cryo-EM structure of the *Shigella* type III needle complex. *PLOS Pathog.* **16**, e1008263 (2020).

15. S. Miletic, D. Fahrenkamp, N. Goessweiner-Mohr, J. Wald, M. Pantel, O. Vesper, V. Kotov, T. C. Marlovits, Substrate-engaged type III secretion system structures reveal gating mechanism for unfolded protein translocation. *Nat. Commun.* **12**, 1546 (2021).

16. M. Yin, Z. Yan, X. Li, Structural insight into the assembly of the type II secretion system pilotin-secretin complex from enterotoxigenic *Escherichia coli*. *Nat. Microbiol.* **3**, 581–587 (2018).

17. S. J. Weaver, D. R. Ortega, M. H. Sazinsky, T. N. Dalia, A. B. Dalia, G. J. Jensen, CryoEM structure of the type IVa pilus secretin required for natural competence in *Vibrio cholerae*. *Nat. Commun.* **11**, 5080 (2020).

18. D. Ghosal, K. W. Kim, H. Zheng, M. Kaplan, H. K. Truchan, A. E. Lopez, I. E. McIntire, J. P. Vogel, N. P. Cianciotto, G. J. Jensen, *In vivo* structure of the *Legionella* type II secretion system by electron cryotomography. *Nat. Microbiol.* **4**, 2101–2108 (2019).

19. J. Spagnuolo, N. Opalka, W. X. Wen, D. Gagic, E. Chabaud, P. Bellini, M. D. Bennett, G. E. Norris, S. A. Darst, M. Russel, J. Rakonjac, Identification of the gate regions in the primary structure of the secretin pIV. *Mol. Microbiol.* **76**, 133–150 (2010).

20. R. A. Dunstan, E. Heinz, L. C. Wijeyewickrema, R. N. Pike, A. W. Purcell, T. J. Evans, J. Praszkier, R. M. Robins-Browne, R. A. Strugnell, K. V. Korotkov, T. Lithgow, Assembly of the type II secretion system such as found in *Vibrio cholerae* depends on the novel Pilotin AspS. *PLOS Pathog.* **9**, e1003117 (2013).

21. B. Douzi, N. T. T. Trinh, S. Michel-Souzy, A. Desmyter, G. Ball, P. Barbier, A. Kosta, E. Durand, K. T. Forest, C. Cambillau, A. Roussel, R. Voulhoux, Unraveling the self-assembly of the *Pseudomonas aeruginosa* XcpQ secretin periplasmic domain provides new molecular insights into type II secretion system secreton architecture and dynamics. *MBio* **8**, e01185-17 (2017).

22. K. V. Korotkov, T. L. Johnson, M. G. Jobling, J. Pruneda, E. Pardon, A. Héroux, S. Turley, J. Steyaert, R. K. Holmes, M. Sandkvist, W. G. J. Hol, Structural and functional studies on the interaction of GspC and GspD in the type II secretion system. *PLOS Pathog.* **7**, e1002228 (2011).

23. R. Van der Meeren, Y. Wen, P. Van Gelder, J. Tommassen, B. Devreese, S. N. Savvides, New insights into the assembly of bacterial secretins: Structural studies of the periplasmic domain of XcpQ from *Pseudomonas aeruginosa*. *J. Biol. Chem.* **288**, 1214–1225 (2013).

24. S. Gu, S. Rehman, X. Wang, V. E. Shevchik, R. W. Pickersgill, Structural and functional insights into the pilotin-secretin complex of the type II secretion system. *PLOS Pathog.* **8**, e1002531 (2012).

25. X. Wang, C. Pineau, S. Gu, N. Guschinskaya, R. W. Pickersgill, V. E. Shevchik, Cysteine scanning mutagenesis and disulfide mapping analysis of arrangement of GspC and GspD protomers within the type 2 secretion system. *J. Biol. Chem.* **287**, 19082–19093 (2012).

26. K. V. Korotkov, J. R. Delarosa, W. G. J. Hol, A dodecameric ring-like structure of the N0 domain of the type II secretin from enterotoxigenic *Escherichia coli*. *J. Struct. Biol.* **183**, 354–362 (2013).

27. K. V. Korotkov, E. Pardon, J. Steyaert, W. G. Hol, Crystal structure of the N-terminal domain of the secretin GspD from ETEC determined with the assistance of a nanobody. *Structure* **17**, 255–265 (2009).

28. C. M. Dade, B. Douzi, C. Cambillau, G. Ball, R. Voulhoux, K. T. Forest, The crystal structure of CbpD clarifies substrate-specificity motifs in chitin-active lytic polysaccharide monooxygenases. *Acta Crystallogr. D Struct. Biol.* **78**, 1064–1078 (2022).

29. C. A. Escobar, B. Douzi, G. Ball, B. Barbat, S. Alphonse, L. Quinton, R. Voulhoux, K. T. Forest, Structural interactions define assembly adapter function of a type II secretion system pseudopilin. *Structure* **29**, 1116–1127.e8 (2021).

30. A. López-Castilla, J.-L. Thomassin, B. Bardiaux, W. Zheng, M. Nivaskumar, X. Yu, M. Nilges, E. H. Egelman, N. Izadi-Pruneyre, O. Francetic, Structure of the calcium-dependent type 2 secretion pseudopilus. *Nat. Microbiol.* **2**, 1686–1695 (2017).

31. N. P. Cianciotto, R. C. White, Expanding role of type II secretion in bacterial pathogenesis and beyond. *Infect. Immun.* **85**, e00014–17 (2017).

32. F. Askarian, S. Uchiyama, H. Masson, H. V. Sørensen, O. Golten, A. C. Bunæs, S. Mekasha, Å. K. Røhr, E. Kommedal, J. A. Ludviksen, M. Ø. Arntzen, B. Schmidt, R. H. Zurich, N. M. van Sorge, V. G. H. Eijsink, U. Krengel, T. E. Mollnes, N. E. Lewis, V. Nizet, G. Vaaje-Kolstad, The lytic polysaccharide monooxygenase CbpD promotes *Pseudomonas aeruginosa* virulence in systemic infection. *Nat. Commun.* **12**, 1230 (2021).

33. B. Douzi, G. Ball, C. Cambillau, M. Tegoni, R. Voulhoux, Deciphering the Xcp *Pseudomonas aeruginosa* type II secretion machinery through multiple interactions with substrates. *J. Biol. Chem.* **286**, 40792–40801 (2011).

34. C. Pineau, N. Guschinskaya, X. Robert, P. Gouet, L. Ballut, V. E. Shevchik, Substrate recognition by the bacterial type II secretion system: More than a simple interaction. *Mol. Microbiol.* **94**, 126–140 (2014).

35. S. Michel-Souzy, B. Douzi, F. Cadoret, C. Raynaud, L. Quinton, G. Ball, R. Voulhoux, Direct interactions between the secreted effector and the T2SS components GspL and GspM reveal a new effector-sensing step during type 2 secretion. *J. Biol. Chem.* **293**, 19441–19450 (2018).

36. K. V. Korotkov, W. G. Hol, Structure of the GspK-GspI-GspJ complex from the enterotoxigenic *Escherichia coli* type 2 secretion system. *Nat. Struct. Mol. Biol.* **15**, 462–468 (2008).

37. B. Douzi, A. Filloux, R. Voulhoux, On the path to uncover the bacterial type II secretion system. *Philos. Trans. R Soc. Lond. B Biol. Sci.* **367**, 1059–1072 (2012).

38. A. de Groot, M. Koster, M. Gérard-Vincent, G. Gerritse, Á.. Lazdunski, J. Tommassen, A. Filloux, Exchange of Xcp (Gsp) secretion machineries between *Pseudomonas aeruginosa* and *Pseudomonas alcaligenes*: Species specificity unrelated to substrate recognition. *J. Bacteriol.* **183**, 959–967 (2001).

39. T. Sana, A. Laubier, S. Bleves, Gene transfer: Conjugation. *Methods Mol. Biol.* **1149**, 17–22 (2014).

40. C. Filip, G. Fletcher, J. L. Wulff, C. F. Earhart, Solubilization of the cytoplasmic membrane of *Escherichia coli* by the ionic detergent sodium-lauryl sarcosinate. *J. Bacteriol.* **115**, 717–722 (1973).

41. A. Fox, D. Haas, C. Reimmann, S. Heeb, A. Filloux, R. Voulhoux, Emergence of secretion-defective sublines of *Pseudomonas aeruginosa* PAO1 resulting from spontaneous mutations in the vfr global regulatory gene. *Appl. Environ. Microbiol.* **74**, 1902–1908 (2008).

42. G. Ball, H. Antelmann, P. R. C. Imbert, M. R. Gimenez, R. Voulhoux, B. Ize, Contribution of the twin arginine translocation system to the exoproteome of *Pseudomonas aeruginosa*. *Sci. Rep.* **6**, 27675 (2016).

43. J. B. Procter, G. M. Carstairs, B. Soares, K. Mourão, T. C. Ofoegbu, D. Barton, L. Lui, A. Menard, N. Sherstnev, D. Roldan-Martinez, S. Duce, D. M. A. Martin, G. J. Barton, Alignment of biological sequences with Jalview. *Methods Mol. Biol.* **2231**, 203–224 (2021).

44. M. Blum, H.Y. Chang, S. Chuguransky, T. Grego, S. Kandasaamy, A. Mitchell, G. Nuka, T. Paysan-Lafosse, M. Qureshi, S. Raj, L. Richardson, G. A. Salazar, L. Williams, P. Bork, A. Bridge, J. Gough, D. H. Haft, I. Letunic, A. Marchler-Bauer, H. Mi, D. A. Natale, M. Necci, C. A. Orengo, A. P. Pandurangan, C. Rivoire, C. J. A. Sigrist, I. Sillitoe, N. Thanki, P. D. Thomas, S. C. E. Tosatto, C. H. Wu, A. Bateman, R. D. Finn, The InterPro protein families and domains database: 20 years on. *Nucleic Acids Res.* **49**, D344–D354 (2021).

45. M. Steinegger, J. Soding, MMseqs2 enables sensitive protein sequence searching for the analysis of massive data sets. *Nat. Biotechnol.* **35**, 1026–1028 (2017).

46. M. Steinegger, J. Soding, Clustering huge protein sequence sets in linear time. *Nat. Commun.* **9**, 2542 (2018).

47. J. Jumper, R. Evans, A. Pritzel, T. Green, M. Figurnov, O. Ronneberger, K. Tunyasuvunakool, R. Bates, A. Žídek, A. Potapenko, A. Bridgland, C. Meyer, S. A. A. Kohl, A. J. Ballard, A. Cowie, B. Romera-Paredes, S. Nikolov, R. Jain, J. Adler, T. Back, S. Petersen, D. Reiman, E. Clancy, M. Zielinski, M. Steinegger, M. Pacholska, T. Berghammer, S. Bodenstein, D. Silver, O. Vinyals, A. W. Senior, K. Kavukcuoglu, P. Kohli, D. Hassabis, Highly accurate protein structure prediction with AlphaFold. *Nature* **596**, 583–589 (2021).

48. M. Varadi, S. Anyango, M. Deshpande, S. Nair, C. Natassia, G. Yordanova, D. Yuan, O. Stroe, G. Wood, A. Laydon, A. Žídek, T. Green, K. Tunyasuvunakool, S. Petersen, J. Jumper, E. Clancy, R. Green, A. Vora, M. Lutfi, M. Figurnov, A. Cowie, N. Hobbs, P. Kohli, G. Kleywegt, E. Birney, D. Hassabis, S. Velankar, AlphaFold protein structure database: Massively expanding

the structural coverage of protein-sequence space with high-accuracy models. *Nucleic Acids Res.* **50**, D439–D444 (2022).

49. D. H. Figurski, D. R. Helinski, Replication of an origin-containing derivative of plasmid RK2 dependent on a plasmid function provided in trans. *Proc. Natl. Acad. Sci. U.S.A.* **76**, 1648–1652 (1979).

50. J. R. Newman, C. Fuqua, Broad-host-range expression vectors that carry the L-arabinose-inducible *Escherichia coli* araBAD promoter and the araC regulator. *Gene* **227**, 197–203 (1999).
